# Supplementary material for: Stacking Model‐Based Classifiers for Dealing With Multiple Sets of Noisy Labels
Source: Biom J. 2025 Mar 12;67(2):e70042. doi: 10.1002/bimj.70042 (PMC11898607; doi:10.1002/bimj.70042)
Supplement: Supplementary file 1 — Supporting Information [file BIMJ-67-e70042-s001.zip › density-based-ensemble-model-main/competitors_real_data/gpml-matlab-master/doc/manual.pdf]

The `GPML` Toolbox version 4.2

Carl Edward Rasmussen & Hannes Nickisch

March 24, 2019

Abstract

The `GPML` toolbox is an Octave 3.2.x and Matlab 7.x implementation of inference and prediction in Gaussian process (GP) models. It implements algorithms discussed in Rasmussen & Williams: Gaussian Processes for Machine Learning , the MIT press, 2006 and Nickisch & Rasmussen: Approximations for Binary Gaussian Process Classification , JMLR, 2008.

The strength of the function lies in its flexibility, simplicity and extensibility. The function is flexible as firstly it allows specification of the properties of the GP through definition of mean function and covariance functions. Secondly, it allows specification of different inference procedures, such as e.g. exact inference and Expectation Propagation (EP). Thirdly it allows specification of likelihood functions e.g. Gaussian or Laplace (for regression) and e.g. cumulative Logistic (for classification). Simplicity is achieved through a single function and compact code. Extensibility is ensured by modular design allowing for easy addition of extension for the already fairly extensive libraries for inference methods, mean functions, covariance functions and likelihood functions.

This document is a technical manual for a developer containing many details. If you are not yet familiar with the `GPML` toolbox, the user documentation and examples therein are a better way to get started.

Contents

1 Gaussian Process Training and Prediction 3

2 The gp Function 5

3 Inference Methods 10
- 3.1 Exact Inference with Gaussian likelihood . . . 11
- 3.2 Laplace's Approximation . . . 12
- 3.3 Expectation Propagation . . . 12
- 3.4 Kullback Leibler Divergence Minimisation . . . 13
- 3.5 Variational Bayes . . . 14
- 3.6 Compatibility Between Inference Methods and Covariance Approximations . . . 14
- 3.7 Sparse Covariance Approximations . . . 15
- 3.8 Grid-Based Covariance Approximations . . . 15
- 3.9 State Space Representation of GPs . . . 16

4 Likelihood Functions 19
- 4.1 Prediction . . . 19
- 4.2 Interface . . . 20
- 4.3 Implemented Likelihood Functions . . . 23
- 4.4 Usage of Implemented Likelihood Functions . . . 23
- 4.5 Compatibility Between Likelihoods and Inference Methods . . . 25
- 4.6 Gaussian Likelihood . . . 25
  - 4.6.1 Exact Inference . . . 26
  - 4.6.2 Laplace's Approximation . . . 26
  - 4.6.3 Expectation Propagation . . . 27
  - 4.6.4 Variational Bayes . . . 27
- 4.7 Warped Gaussian Likelihood . . . 27
- 4.8 Gumbel Likelihood . . . 28
- 4.9 Laplace Likelihood . . . 29
- 4.10 Student's t Likelihood . . . 32
- 4.11 Cumulative Logistic Likelihood . . . 32
- 4.12 GLM Likelihoods: Poisson, Negative Binomial, Weibull, Gamma, Exponential, Inverse Gaussian and Beta . . . 33
  - 4.12.1 Inverse Link Functions . . . 33
  - 4.12.2 Poisson Likelihood . . . 35
  - 4.12.3 Weibull Likelihood . . . 35
  - 4.12.4 Gamma Likelihood . . . 36
  - 4.12.5 Exponential Likelihood . . . 36
  - 4.12.6 Inverse Gaussian Likelihood . . . 36
  - 4.12.7 Log Gaussian Likelihood . . . 36
  - 4.12.8 Beta Likelihood . . . 37

5 Mean Functions 38
- 5.1 Interface . . . 38
- 5.2 Implemented Mean Functions . . . 39
- 5.3 Usage of Implemented Mean Functions . . . 39

6 Covariance Functions 41
- 6.1 Interface . . . 41
- 6.2 Implemented Covariance Functions . . . 44

6.3 Usage of Implemented Covariance Functions 45

7 Hyperpriors 47
7.1 Interface 47
7.2 Implemented Hyperpriors 49
7.3 Usage of Implemented Hyperpriors 49

1 Gaussian Process Training and Prediction

The `gpml` toolbox contains a single user function `gp` described in section 2. In addition there are a number of supporting structures and functions which the user needs to know about, as well as an internal convention for representing the posterior distribution, which may not be of direct interest to the casual user.

Inference Methods: An inference method is a function which computes the (approximate) posterior, the (approximate) negative log marginal likelihood and its partial derivatives w.r.t.. the hyperparameters, given a model specification (i.e., GP mean and covariance functions and a likelihood function) and a data set. Inference methods are discussed in section 3. New inference methods require a function providing the desired inference functionality and possibly extra functionality in the likelihood functions applicable.

Hyperparameters: The hyperparameters is a struct controlling the properties of the model, i.e.. the GP mean and covariance function and the likelihood function. The hyperparameters is a struct with the three fields `mean`, `cov` and `lik`, each of which is a vector. The number of elements in each field must agree with number of hyperparameters in the specification of the three functions they control (below). If a field is either empty or non-existent it represents zero hyperparameters. When working with FITC approximate inference, the inducing inputs `xu` can also be treated as hyperparameters for some common stationary covariances.

Hyperparameter Prior Distributions: When optimising the marginal likelihood w.r.t. hyperparameters, it is sometimes useful to softly constrain the hyperparameters by means of prior knowledge. A `prior` is a probability distribution over individual or a group of hyperparameters, section 7.

Likelihood Functions: The likelihood function specifies the form of the likelihood of the GP model and computes terms needed for prediction and inference. For inference, the required properties of the likelihood depend on the inference method, including properties necessary for hyperparameter learning, section 4.

Mean Functions: The mean function is a cell array specifying the GP mean. It computes the mean and its derivatives w.r.t.. the part of the hyperparameters pertaining to the mean. The cell array allows flexible specification and composition of mean functions, discussed in section 5. The default is the zero function.

Covariance Functions: The covariance function is a cell array specifying the GP covariance function. It computes the covariance and its derivatives w.r.t.. the part of the hyperparameters pertaining to the covariance function. The cell array allows flexible specification and composition of covariance functions, discussed in section 6.

Inference methods, see section 3, compute (among other things) an approximation to the posterior distribution of the latent variables f_i associated with the training cases, $i = 1, \dots, n$. This approximate posterior is assumed to be Gaussian, and is communicated via a struct `post` with the fields `post.alpha`, `post.sW` and `post.L`. Often, starting from the Gaussian prior $p(f) = \mathcal{N}(f|m, K)$ the approximate posterior admits the form

$$q(f|\mathcal{D}) \;=\; \mathcal{N}\big(f|\mu = m + K\alpha,\; V = (K^{-1} + W)^{-1}\big), \quad \text{where} \quad W \; \text{diagonal with} \quad W_{ii} = s_i^2. \qquad (1)$$

In such cases, the entire posterior can be computed from the two vectors `post.alpha` and `post.sW`; the inference method may optionally also return $L = \text{chol}(\text{diag}(s)K\,\text{diag}(s) + I)$.
If on the other hand the posterior doesn't admit the above form, then `post.L` returns the matrix

$\mathsf{L} = -(\mathsf{K} + W^{-1})^{-1}$ (and `post.sW` is unused). In addition, a sparse representation of the posterior may be used, in which case the non-zero elements of the `post.alpha` vector indicate the active entries.

2 The gp Function

The gp function is typically the only function the user would directly call.

5a ⟨gp.m 5a⟩≡

```
function [varargout] = gp(hyp, inf, mean, cov, lik, x, y, xs, ys)
⟨gp function help 5b⟩
⟨initializations 6b⟩
⟨inference 7c⟩
if nargin==7                                                  % if no test cases are provided
  varargout = {nlZ, dnlZ, post};    % report -log marg lik, derivatives and post
else
  ⟨compute test predictions 8⟩
end
```

It offers facilities for training the hyperparameters of a GP model as well as predictions at unseen inputs as detailed in the following help.

5b ⟨gp function help 5b⟩≡ (5a)

```
% Gaussian Process inference and prediction. The gp function provides a
% flexible framework for Bayesian inference and prediction with Gaussian
% processes for scalar targets, i.e. both regression and binary
% classification. The prior is Gaussian process, defined through specification
% of its mean and covariance function. The likelihood function is also
% specified. Both the prior and the likelihood may have hyperparameters
% associated with them.
%
% Two modes are possible: training or prediction: if no test cases are
% supplied, then the negative log marginal likelihood and its partial
% derivatives w.r.t. the hyperparameters is computed; this mode is used to fit
% the hyperparameters. If test cases are given, then the test set predictive
% probabilities are returned. Usage:
%
%   training: [nlZ dnlZ          ] = gp(hyp, inf, mean, cov, lik, x, y);
% prediction: [ymu ys2 fmu fs2   ] = gp(hyp, inf, mean, cov, lik, x, y, xs);
%         or: [ymu ys2 fmu fs2 lp] = gp(hyp, inf, mean, cov, lik, x, y, xs, ys);
%
% where:
%
%   hyp      struct of column vectors of mean/cov/lik hyperparameters
%   inf      function specifying the inference method
%   mean     prior mean function
%   cov      prior covariance function
%   lik      likelihood function
%   x        n by D matrix of training inputs
%   y        column vector of length n of training targets
%   xs       ns by D matrix of test inputs
%   ys       column vector of length nn of test targets
%
%   nlZ      returned value of the negative log marginal likelihood
%   dnlZ     struct of column vectors of partial derivatives of the negative
%               log marginal likelihood w.r.t. mean/cov/lik hyperparameters
%   ymu      column vector (of length ns) of predictive output means
%   ys2      column vector (of length ns) of predictive output variances
%   fmu      column vector (of length ns) of predictive latent means
%   fs2      column vector (of length ns) of predictive latent variances
%   lp       column vector (of length ns) of log predictive probabilities
%
```

```
%   post     struct representation of the (approximate) posterior
%            3rd output in training mode or 6th output in prediction mode
%            can be reused in prediction mode gp(.., cov, lik, x, post, xs,..)
%
% See also infMethods.m, meanFunctions.m, covFunctions.m, likFunctions.m.
%
⟨gpml copyright 6a⟩
```

6a ⟨gpml copyright 6a⟩≡ (5b 10 11 20 23 25 38 40 41 45 47 50)

```
% Copyright (c) by Carl Edward Rasmussen and Hannes Nickisch, 2019-03-24.
%                                        File automatically generated using noweb.
```

Depending on the number of input parameters, gp knows whether it is operated in training or in prediction mode. The highlevel structure of the code is as follows. After some initialisations, we perform inference and decide whether test set predictions are needed or only the result of the inference is demanded.

6b ⟨initializations 6b⟩≡ (5a)

```
⟨minimalist usage 6c⟩
⟨multivariate output by recursion 6d⟩
⟨process input arguments 7a⟩
⟨check hyperparameters 7b⟩
```

If the number of input arguments is incorrect, we echo a minimalist usage and return.

6c ⟨minimalist usage 6c⟩≡ (6b)

```
if nargin<7 || nargin>9
  disp('Usage: [nlZ dnlZ          ] = gp(hyp, inf, mean, cov, lik, x, y);')
  disp('   or: [ymu ys2 fmu fs2   ] = gp(hyp, inf, mean, cov, lik, x, y, xs);')
  disp('   or: [ymu ys2 fmu fs2 lp] = gp(hyp, inf, mean, cov, lik, x, y, xs, ys);')
  return
end
```

If there is more than a single output dimension in y, we call multiple instances of gp with shared hyperparameters and settings each computing the corresponding result with scalar output.

6d ⟨multivariate output by recursion 6d⟩≡ (6b)

```
if size(y,2)>1      % deal with (independent) multivariate output y by recursing
  d = size(y,2); varargout = cell(nargout,1); out = cell(nargout,1);  % allocate
  for i=1:d
    in = {hyp, inf, mean, cov, lik, x, y(:,i)};
    if nargin>7, in = {in{:}, xs}; end
    if nargin>8, in = {in{:}, ys(:,i)}; end
    if i==1, [varargout{:}] = gp(in{:});    % perform inference for dimension ..
    else            [out{:}] = gp(in{:});            % .. number i in the output
      if nargin==7, no = 2;
        varargout{1} = varargout{1} + out{1};                           % sum nlZ
        if nargout>1                                                   % sum dnlZ
          varargout{2} = vec2any(hyp,any2vec(varargout{2})+any2vec(out{2}));
        end
      else no = 5;                          % concatenate ymu ys2 fmu fs2 lp
        for j=1:min(nargout,no), varargout{j} = [varargout{j},out{j}]; end
      end
      if nargout>no                                           % concatenate post
        if i==2, varargout{no+1} = {varargout{no+1},out{no+1}};
        else varargout{no+1} = {varargout{no+1}{:},out{no+1}}; end
      end
    end
  end, return                                       % return to end the recursion
end
```

Next, we set some useful default values for empty arguments, and convert `inf` and `lik` to function handles and `mean` and `cov` to cell arrays if necessary. Initialize variables.

7a ⟨process input arguments 7a⟩≡ (6b)

```
if isempty(mean), mean = {@meanZero}; end                          % set default mean
if ischar(mean) || isa(mean, 'function_handle'), mean = {mean}; end  % make cell
if isempty(cov), error('Covariance function cannot be empty'); end  % no default
if ischar(cov) || isa(cov,'function_handle'), cov  = {cov};  end     % make cell
cstr = cov{1}; if isa(cstr,'function_handle'), cstr = func2str(cstr); end
if (strcmp(cstr,'covFITC') || strcmp(cstr,'apxSparse')) && isfield(hyp,'xu')
  cov{3} = hyp.xu;                                                        %use hyp.xu
end
if isempty(inf), inf = {@infGaussLik}; end        % set default inference method
if ischar(inf),  inf = str2func(inf);  end        % convert into function handle
if ischar(inf) || isa(inf,'function_handle'), inf = {inf};  end      % make cell
istr = inf{1}; if isa(istr,'function_handle'), istr = func2str(istr); end
if strcmp(istr,'infPrior')
  istr = inf{2}; if isa(istr,'function_handle'), istr = func2str(istr); end
end
if isempty(lik), lik = {@likGauss}; end                         % set default lik
if ischar(lik) || isa(lik,'function_handle'), lik = {lik};  end      % make cell
lstr = lik{1}; if isa(lstr,'function_handle'), lstr = func2str(lstr); end

D = size(x,2);
if strncmp(cstr,'covGrid',7) || strcmp(cstr,'apxGrid') % only some inf* possible
  D = 0; xg = cov{3}; p = numel(xg); for i=1:p, D = D+size(xg{i},2); end  % dims
end
```

Check that the sizes of the hyperparameters supplied in `hyp` match the sizes expected. The three parts `hyp.mean`, `hyp.cov` and `hyp.lik` are checked separately, and define empty entries if they don't exist.

7b ⟨check hyperparameters 7b⟩≡ (6b)

```
if ~isfield(hyp,'mean'), hyp.mean = []; end        % check the hyp specification
if eval(feval(mean{:})) ~= numel(hyp.mean)
  error('Number of mean function hyperparameters disagree with mean function')
end
if ~isfield(hyp,'cov'), hyp.cov = []; end
if eval(feval(cov{:})) ~= numel(hyp.cov)
  error('Number of cov function hyperparameters disagree with cov function')
end
if ~isfield(hyp,'lik'), hyp.lik = []; end
if eval(feval(lik{:})) ~= numel(hyp.lik)
  error('Number of lik function hyperparameters disagree with lik function')
end
```

Inference is performed by calling the desired inference method `inf`. In training mode, we accept a failure of the inference method (and issue a warning), since during hyperparameter learning, hyperparameters causing a numerical failure may be attempted, but the `minimize` function may gracefully recover from this. During prediction, failure of the inference method is an error.

7c ⟨inference 7c⟩≡ (5a)

```
try                                                  % call the inference method
  % issue a warning if a classification likelihood is used in conjunction with
  % labels different from +1 and -1
  if strcmp(lstr,'likErf') || strcmp(lstr,'likLogistic')
    if ~isstruct(y)
      uy = unique(y);
      if any( uy~=+1 & uy~=-1 )
```

```
        warning('You try classification with labels different from {+1,-1}')
      end
    end
  end
  if nargin>7   % compute marginal likelihood and its derivatives only if needed
    if isstruct(y)
      post = y;            % reuse a previously computed posterior approximation
    else
      post = feval(inf{:}, hyp, mean, cov, lik, x, y);
    end
  else
    if nargout<=1
      [post nlZ] = feval(inf{:}, hyp, mean, cov, lik, x, y); dnlZ = {};
    else
      [post nlZ dnlZ] = feval(inf{:}, hyp, mean, cov, lik, x, y);
    end
  end
catch
  msgstr = lasterr;
  if nargin>7, error('Inference method failed [%s]', msgstr); else
    warning('Inference method failed [%s] .. attempting to continue',msgstr)
    varargout = {NaN, vec2any(hyp,zeros(numel(any2vec(hyp)),1))}; return % go on
  end
end
```

We copy the already computed negative log marginal likelihood to the first output argument, and if desired report its partial derivatives w.r.t. the hyperparameters if running in inference mode.

Predictions are computed in a loop over small batches to avoid memory problems for very large test sets.

8 ⟨compute test predictions 8⟩≡ (5a)

```
alpha = post.alpha; L = post.L; sW = post.sW;
if issparse(alpha)                    % handle things for sparse representations
  nz = alpha ~= 0;                                  % determine nonzero indices
  if issparse(L), L = full(L(nz,nz)); end      % convert L and sW if necessary
  if issparse(sW), sW = full(sW(nz)); end
else nz = true(size(alpha,1),1); end                % non-sparse representation
if isempty(L)                          % in case L is not provided, we compute it
  K = feval(cov{:}, hyp.cov, x(nz,:));
  L = chol(eye(sum(nz))+sW*sW'.*K);
end
%verify whether L contains valid Cholesky decomposition or something different
Lchol = isnumeric(L) && all(all(tril(L,-1)==0)&diag(L)'>0&isreal(diag(L))');
ns = size(xs,1);                                        % number of data points
if strncmp(cstr,'apxGrid',7), xs = apxGrid('idx2dat',cov{3},xs); end  % expand
nperbatch = 1000;                         % number of data points per mini batch
nact = 0;                       % number of already processed test data points
ymu = zeros(ns,1); ys2 = ymu; fmu = ymu; fs2 = ymu; lp = ymu;   % allocate mem
while nact<ns               % process minibatches of test cases to save memory
  id = (nact+1):min(nact+nperbatch,ns);               % data points to process
  ⟨make predictions 9⟩
  nact = id(end);          % set counter to index of last processed data point
end
if nargin<9
  varargout = {ymu, ys2, fmu, fs2, [], post};        % assign output arguments
else
  varargout = {ymu, ys2, fmu, fs2, lp, post};
end
```

In every iteration of the above loop, we compute the predictions for all test points of the batch.

9 ⟨make predictions 9⟩≡ (8)

```
kss = feval(cov{:}, hyp.cov, xs(id,:), 'diag');              % self-variance
if strcmp(cstr,'covFITC') || strcmp(cstr,'apxSparse')    % cross-covariances
  Ks = feval(cov{:}, hyp.cov, x, xs(id,:)); Ks = Ks(nz,:); % res indep. of x
else
  Ks = feval(cov{:}, hyp.cov, x(nz,:), xs(id,:));        % avoid computation
end
ms = feval(mean{:}, hyp.mean, xs(id,:));
N = size(alpha,2);  % number of alphas (usually 1; more in case of sampling)
Fmu = repmat(ms,1,N) + Ks'*full(alpha(nz,:));        % conditional mean fs|f
fmu(id) = sum(Fmu,2)/N;                                   % predictive means
if Lchol    % L contains chol decomp => use Cholesky parameters (alpha,sW,L)
  V  = L'\(repmat(sW,1,length(id)).*Ks);
  fs2(id) = kss - sum(V.*V,1)';                       % predictive variances
else                % L is not triangular => use alternative parametrisation
  if isnumeric(L), LKs = L*Ks; else LKs = L(Ks); end    % matrix or callback
  fs2(id) = kss + sum(Ks.*LKs,1)';                    % predictive variances
end
fs2(id) = max(fs2(id),0);   % remove numerical noise i.e. negative variances
Fs2 = repmat(fs2(id),1,N);     % we have multiple values in case of sampling
if nargin<9
  [Lp, Ymu, Ys2] = feval(lik{:},hyp.lik,[],   Fmu(:),Fs2(:));
else
  Ys = repmat(ys(id),1,N);
  [Lp, Ymu, Ys2] = feval(lik{:},hyp.lik,Ys(:),Fmu(:),Fs2(:));
end
lp(id)  = sum(reshape(Lp, [],N),2)/N;    % log probability; sample averaging
ymu(id) = sum(reshape(Ymu,[],N),2)/N;          % predictive mean ys|y and ..
ys2(id) = sum(reshape(Ys2,[],N),2)/N;                          % .. variance
```

3 Inference Methods

Inference methods are responsible for computing the (approximate) posterior `post`, the (approximate) negative log marginal likelihood `nlZ` and its partial derivatives `dnlZ` w.r.t. the hyperparameters `hyp`. The arguments to the function are hyperparameters `hyp`, mean function `mean`, covariance function `cov`, likelihood function `lik` and training data `x` and `y`. Several inference methods are implemented and described this section.

10 ⟨infMethods.m 10⟩≡

```
% Inference methods: Compute the (approximate) posterior for a Gaussian process.
% Methods currently implemented include:
%
%   infGaussLik      Exact inference (only possible with Gaussian likelihood)
%   infLaplace       Laplace's Approximation
%   infEP            Expectation Propagation
%   infVB            Variational Bayes Approximation
%   infKL            Kullback-Leibler optimal Approximation
%
%   infMCMC     Markov Chain Monte Carlo and Annealed Importance Sampling
%               We offer two samplers.
%                 - hmc: Hybrid Monte Carlo
%                 - ess: Elliptical Slice Sampling
%               No derivatives w.r.t. to hyperparameters are provided.
%
%   infLOO      Leave-One-Out predictive probability and Least-Squares Approxim.
%   infPrior    Perform inference with hyperparameter prior.
%
% The interface to the approximation methods is the following:
%
%   function [post nlZ dnlZ] = inf..(hyp, mean, cov, lik, x, y)
%
% where:
%
%   hyp      is a struct of hyperparameters
%   mean     is the name of the mean function       (see meanFunctions.m)
%   cov      is the name of the covariance function (see covFunctions.m)
%   lik      is the name of the likelihood function (see likFunctions.m)
%   x        is a n by D matrix of training inputs
%   y        is a (column) vector (of size n) of targets
%
%   nlZ      is the returned value of the negative log marginal likelihood
%   dnlZ     is a (column) vector of partial derivatives of the negative
%               log marginal likelihood w.r.t. each hyperparameter
%   post     struct representation of the (approximate) posterior containing
%     alpha  is a (sparse or full column vector) containing inv(K)*(mu-m),
%               where K is the prior covariance matrix, m the prior mean,
%               and mu the approx posterior mean
%     sW     is a (sparse or full column) vector containing diagonal of sqrt(W)
%               the approximate posterior covariance matrix is inv(inv(K)+W)
%     L      is a (sparse or full) triangular matrix, L = chol(sW*K*sW+eye(n)),
%               or a full matrix, L = -inv(K+inv(W)),
%               or a function L(A) of a matrix A such that -(K+inv(W))*L(A) = A
%
% Usually, the approximate posterior to be returned admits the form
% N(mu=m+K*alpha, V=inv(inv(K)+W)), where alpha is a vector and W is diagonal.
%
% For more information on the individual approximation methods and their
```

```
% implementations, see the separate inf??.m files. See also gp.m.
%
⟨gpml copyright 6a⟩
```

Not all inference methods are compatible with all likelihood functions, e.g.. exact inference is only possible with Gaussian likelihood. In order to perform inference, each method needs various properties of the likelihood functions, section 4.

3.1 Exact Inference with Gaussian likelihood

For Gaussian likelihoods, GP inference reduces to computing mean and covariance of a multivariate Gaussian which can be done exactly by simple matrix algebra. The program `inf/infExact.m` does exactly this. If it is called with a likelihood function other than the Gaussian, it issues an error. The Gaussian posterior $q(f|\mathcal{D}) = \mathcal{N}(f|\mu, V)$ is exact.

11 ⟨inf/infGaussLik.m 11⟩≡

```
function [post nlZ dnlZ] = infGaussLik(hyp, mean, cov, lik, x, y, opt)

% Exact inference for a GP with Gaussian likelihood.
%
% Compute a parametrization of the posterior, the negative log marginal
% likelihood and its derivatives w.r.t. the hyperparameters. The function takes
% a specified covariance function (see covFunctions.m) and likelihood function
% (see likFunctions.m), and is designed to be used with gp.m.
%
⟨gpml copyright 6a⟩
%
% See also infMethods.m, cov/apx.m.

if nargin<7, opt = []; end                          % make sure parameter exists
if iscell(lik), likstr = lik{1}; else likstr = lik; end
if ~ischar(likstr), likstr = func2str(likstr); end
if ~strcmp(likstr,'likGauss')               % NOTE: no explicit call to likGauss
  error('Exact inference only possible with Gaussian likelihood');
end

[n, D] = size(x);
[m,dm] = feval(mean{:}, hyp.mean, x);           % evaluate mean vector and deriv
sn2 = exp(2*hyp.lik); W = ones(n,1)/sn2;            % noise variance of likGauss
K = apx(hyp,cov,x,opt);                        % set up covariance approximation
[ldB2,solveKiW,dW,dhyp,post.L] = K.fun(W); % obtain functionality depending on W

alpha = solveKiW(y-m);
post.alpha = K.P(alpha);                       % return the posterior parameters
post.sW = sqrt(W);                              % sqrt of noise precision vector
if nargout>1                               % do we want the marginal likelihood?
  nlZ = (y-m)'*alpha/2 + ldB2 + n*log(2*pi*sn2)/2;    % -log marginal likelihood
  if nargout>2                                         % do we want derivatives?
    dnlZ = dhyp(alpha); dnlZ.mean = -dm(alpha);
    dnlZ.lik = -sn2*(alpha'*alpha) - 2*sum(dW)/sn2 + n;
  end
end
```

3.2 Laplace's Approximation

For differentiable likelihoods, Laplace's approximation, approximates the posterior by a Gaussian centered at its mode and matching its curvature `inf/infLaplace.m`.

More concretely, the mean of the posterior $q(f|\mathcal{D}) = \mathcal{N}\big(f|\mu, V\big)$ is – defining $\ell_i(f_i) = \ln p(y_i|f_i)$ and $\ell(f) = \sum_{i=1}^n \ell_i(f_i)$ – given by

$$\mu = \arg\min_f \phi(f), \text{ where } \phi(f) = \frac{1}{2}(f-m)^\top K^{-1}(f-m) - \ell(f) \stackrel{c}{=} -\ln[p(f)p(y|f)], \tag{2}$$

which we abbreviate by $\mu \leftarrow \mathcal{L}(\ell)$. The curvature $\frac{\partial^2\phi}{\partial f f^\top} = K^{-1} + W$ with $W_{ii} = -\frac{\partial^2}{\partial f_i^2}\ln p(y_i|f_i)$ serves as precision for the Gaussian posterior approximation $V = (K^{-1}+W)^{-1}$ and the marginal likelihood $Z = \int p(f)p(y|f)df$ is approximated by $Z \approx Z_{LA} = \int \tilde{\phi}(f)df$ where we use the 2nd order Taylor expansion at the mode μ given by $\tilde{\phi}(f) = \phi(\mu) + \frac{1}{2}(f-\mu)^\top V^{-1}(f-\mu) \approx \phi(f)$.

Laplace's approximation needs derivatives up to third order for the mode fitting procedure (Newton method)

$$d_k = \frac{\partial^k}{\partial f^k}\log p(y|f), \quad k = 0, 1, 2, 3$$

and

$$d_k = \frac{\partial}{\partial \rho_i}\frac{\partial^k}{\partial f^k}\log p(y|f), \quad k = 0, 1, 2$$

evaluated at the latent location f and observed value y. The likelihood calls (see section 4)

- `[d0, d1, d2, d3] = lik(hyp, y, f, [], 'infLaplace')`

and

- `[d0, d1, d2] = lik(hyp, y, f, [], 'infLaplace', i)`

return exactly these values.

3.3 Expectation Propagation

The basic idea of Expectation Propagation (EP) as implemented in `inf/infEP.m`. is to replace the non-Gaussian likelihood terms $p(y_i|f_i)$ by Gaussian functions $t(f_i;\nu_i,\tau_i) = \exp(\nu_i f_i - \frac{1}{2}\tau_i f_i^2)$ and to adjust the natural parameters ν_i, τ_i such that the following identity holds:

$$\frac{1}{Z_{t,i}}\int f^k q_{-i}(f)\cdot t(f;\nu_i,\tau_i)df = \frac{1}{Z_{p,i}}\int f^k q_{-i}(f)\cdot p(y_i|f)df, \quad k = 1, 2$$

with the so-called cavity distributions $q_{-i}(f) = \mathcal{N}(f|m,K)\prod_{j\neq i} t(f_j;\nu_j,\tau_j) \propto \mathcal{N}(f|\mu,V)/t(f_i;\nu_i,\tau_i)$ equal to the posterior divided by the ith Gaussian approximation function and the two normalisers $Z_{t,i} = \int q_{-i}(f)\cdot t(f_i;\nu_i,\tau_i)df$ and $Z_{p,i} = \int q_{-i}(f)\cdot p(y_i|f_i)df$. The moment matching corresponds to minimising the following local KL-divergence

$$\nu_i, \tau_i = \arg\min_{\nu,\tau} KL[q_{-i}(f)p(y_i|f_i)/Z_{p,i} || q_{-i}(f)t(f_i;\nu,\tau)/Z_{t,i}].$$

In order to apply the moment matching steps in a numerically safe way, EP requires the deriviatives of the expectations w.r.t. the Gaussian mean parameter μ

$$d_k = \frac{\partial^k}{\partial \mu^k}\log\int p(y|f)\mathcal{N}(f|\mu,\sigma^2)df, \quad k = 0, 1, 2$$

and the ith likelihood hyperparameter ρ_i

$$d = \frac{\partial}{\partial \rho_i} \log \int p(y|f) \mathcal{N}(f|\mu, \sigma^2) df$$

which can be obtained by the likelihood calls (see section 4)

- `[d0, d1, d2] = lik(hyp, y, mu, s2, 'infEP')`

and

- `d = lik(hyp, y, mu, s2, 'infEP', i)`.

3.4 Kullback Leibler Divergence Minimisation

Another well known approach to approximate inference implemented `inf/infKL.m` in attempts to directly find the closest Gaussian $q(f|\mathcal{D}) = \mathcal{N}(f|\mu, V)$ to the exact posterior $p(f|\mathcal{D})$ w.r.t. to some proximity measure or equivalently to maximise a lower bound $Z(\mu, V)$ to the marginal likelihood Z as described in Nickisch & Rasmussen Approximations for Binary Gaussian Process Classification, JMLR, 2008. In particular, one minimises $\mathrm{KL}\left(\mathcal{N}(f|\mu, V)||p(f|\mathcal{D})\right)$ which amounts to minimising $-\ln Z(\mu, V)$ as defined by:

$$\begin{aligned}
-\ln Z &= -\ln \int p(f)p(y|f)df = -\ln \int q(f|\mathcal{D}) \frac{p(f)}{q(f|\mathcal{D})} p(y|f)df \\
&\overset{\text{Jensen}}{\leqslant} \int q(f|\mathcal{D}) \ln \frac{q(f|\mathcal{D})}{p(f)} df - \int q(f|\mathcal{D}) \ln p(y|f) df =: -\ln Z(\mu, V) \\
&= \mathrm{KL}\left(\mathcal{N}(f|\mu, V)||\mathcal{N}(f|m, K)\right) - \sum_{i=1}^{n} \int \mathcal{N}(f_i|\mu_i, v_{ii}) \ln p(y_i|f_i) df_i, \; v_{ii} = [V]_{ii} \\
&= \frac{1}{2}\left(\mathrm{tr}(VK^{-1} - I) - \ln |VK^{-1}|\right) + \frac{1}{2}(\mu - m)^\top K^{-1}(\mu - m) - \sum_{i=1}^{n} \ell^{\mathrm{KL}}(\mu_i, v_{ii})
\end{aligned}$$

where $\ell_v^{\mathrm{KL}}(\mu_i) = \int \mathcal{N}(f_i|\mu_i, v_{ii}) \ell_i(f_i) df_i$ is the convolution of the log likelihood ℓ_i with the Gaussian $\mathcal{N}$ and $v = \mathrm{dg}(V)$. Equivalently, one can view ℓ^{KL} as a smoothed version of ℓ with univariate smoothing kernel $\mathcal{N}$.

From Challis & Barber Concave Gaussian Variational Approximations for Inference in Large Scale Bayesian Linear Models, AISTATS, 2011 we know that the mapping $(\mu, L) \mapsto -\ln Z(\mu, L^\top L)$ is jointly convex whenever the likelihoods $f_i \mapsto \mathbb{P}(y_i|f_i)$ are log concave. In particular, this implies that every $(\mu_i, s_i) \mapsto -\ell^{\mathrm{KL}}(\mu_i, s_i^2)$ is jointly convex.

We use an optimisation algorithm similar to EP (section 3.3) where we minimise the local KL-divergence the other way round $\mu_i, s_i = \arg\min_{\mu,s} \mathrm{KL}[\mathcal{N}(f|\mu, s^2)||q_{-i}(f)p(y_i|f_i)/Z_{p,i}]$. This view was brought forward by Tom Minka Convex Divergence measures and message passing, MSR-TR, 2005. The KL minimisation constitutes a jointly convex 2d optimisation problem solved by `klmin` using a scaled Newton approach which is included as a sub function in `inf/infKL.m`. The smoothed likelihood $\ell^{\mathrm{KL}}(\mu_i, v_{ii})$ is implemented as a meta likelihood in `likKL`; it uses Gaussian-Hermite quadrature to compute the required integrals. Note that – as opposed to EP – Gaussian-Hermite quadrature is appropriate since we integrate against the $\ln \mathbb{P}(y_i|f_i)$ (which can be well approximated by a polynomial) instead of $\mathbb{P}(y_i|f_i)$ itself. The algorithm is – again unlike EP – provably convergent for log-concave likelihoods (e.g. `likGauss`, `likLaplace`, `likSech2`, `likLogistic`, `likPoisson`) since it can be regarded as coordinate descent with guaranteed decrease in the objective in every step. Due to the complex update computations, `infKL` can be quite slow although it has the same $\mathcal{O}(n^3)$ asymptotic complexity as EP and Laplace.

3.5 Variational Bayes

One can drive the bounding even further by means of local quadratic lower bounds to the log likelihood $\ell(f) = \ln p(y|f)$. Suppose that we use a super-Gaussian likelihood $p(y|f)$ i.e. likelihoods that can be lower bounded by Gaussians of any width w (e.g. `likLaplace`, `likT`, `likLogistic`, `likSech2`). Formally, that means that there are $b, z \in \mathbb{R}$ such that

$$\rho(f) = \ln p(y|f - z) - bf$$

is symmetric and $\sqrt{f} \mapsto \rho(f)$ is a convex function for all $f \geqslant 0$. As a result, we obtain the following exact representation of the likelihood

$$\ell(f) \quad = \quad \ln p(y|f) = \max_{w>0} \left((b + wz)f - \frac{wf^2}{2} - \frac{1}{2}h(\gamma) \right),$$

which can be derived by convex duality and assuming the likelihoods to be super-Gaussian. Details can be found in papers by Palmer et al. Variational EM Algorithms for Non-Gaussian Latent Variable Models, NIPS, 2006 and Nickisch & Seeger Convex Variational Bayesian Inference for Large Scale Generalized Linear Models, ICML, 2009.

The bottom line is that we can treat the variational bounding as a sequence of Laplace approximations with the "variational Bayes" log likelihood

$$\ell^{\text{VB}}(f_i) = \ell(g_i) + b_i(f_i - g_i), \text{ g} = \text{sgn}(\text{f} - \text{z}) \odot \sqrt{(\text{f} - \text{z})^2 + \text{v}} + \text{z}$$

instead of the usual likelihood $\ell(f_i) = \ln p(y_i|f_i)$ i.e. we solve $\boldsymbol{\mu} \leftarrow \mathcal{L}(\ell_{\text{v}}^{\text{VB}})$ instead of $\boldsymbol{\mu} \leftarrow \mathcal{L}(\ell)$. See section 3.2. In the code of `inf/infVB.m`, the likelihood is implemented in the function `likVB`.

At the end, the optimal value of W can be obtained analytically via $w_i = |b_i - \ell'(g_i)|/|g_i - z_i|$.

For the minimisation in `inf/infVB.m`, we use a provably convergent double loop algorithm, where in the inner loop a nonlinear least squares problem (convex for log-concave likelihoods) is solved using `inf/infLaplace.m` such that $\boldsymbol{\mu} \leftarrow \mathcal{L}(\ell_{\text{v}}^{\text{VB}})$ and in the outer loop, we compute $\text{v} \leftarrow \text{dg}((\text{K}^{-1} + \text{W})^{-1})$. The only requirement to the likelihood function is that it returns the values z and b required by the bound which are delivered by the call (see section 4)

- `[b,z] = lik(hyp, y, [], ga, 'infVB')`

The negative marginal likelihood upper bound $-\ln Z_{\text{VB}}$ is obtained by integrating the prior times the exact representation of the likelihood

$$p(y|f) = \max_{\gamma>0} q(y|f,\gamma), \; q(y|f,\gamma) = \mathcal{N}(f|\nu,\gamma) \exp\left(-\frac{h(\gamma)}{2} - \frac{\nu^2}{2\gamma}\right)\sqrt{2\pi\gamma}, \; \gamma = \frac{1}{w}, \; \nu = b\gamma + z$$

w.r.t. the latent variables f yielding

$$\begin{aligned} -\ln Z_{\text{VB}} &= -\ln \int \mathcal{N}(\text{f}|\text{m}, \text{K}) \prod_{i=1}^{n} q_i(y_i|f_i, \gamma_i)\text{df} \\ &= -\ln \mathcal{N}(\text{m}|\boldsymbol{\nu}, \text{K} + \boldsymbol{\Gamma}) + \frac{1}{2}\left(h(\boldsymbol{\gamma}) - \text{w}^\top \boldsymbol{\nu}^2 - 1^\top \ln 2\pi\boldsymbol{\gamma}\right). \end{aligned}$$

3.6 Compatibility Between Inference Methods and Covariance Approximations

Another kind of approximation is needed to render an inference method scalable. We have two approximation schemes which in fact approximate the covariance to make it amenable to large number of training data points. The following table shows the compatibility between some inference methods and two major groups of covariance approximations we will discuss in the next two sections.

Approximation \ Inference	Exact	Laplace	VB	EP	KL, MCMC, LOO	Implementation
	`infGaussLik`	`infLaplace`	`infVB`	`infEP`	`inf{KL,MCMC,LOO}`	
variational free energy (VFE)	✓	✓	✓			`apxSparse`, `opt.s=0.0`
fully independent training conditionals (FITC)	✓	✓	✓	✓		`apxSparse`, `opt.s=1.0`
hybrid between VFE and FITC, $s_0 \in [0,1]$	✓	✓	✓			`apxSparse`, `opt.s=s0`
Kronecker/Toeplitz/BTTB grid (KISS-GP)	✓	✓	✓			`apxGrid`
State space representation	✓	✓	✓	(✓, ADF)		`apxState`

3.7 Sparse Covariance Approximations

One of the main problems with GP models is the high computational load for inference computations. In a setting with n training points x, exact inference with Gaussian likelihood requires $O(n^3)$ effort; approximations like Laplace or EP consist of a sequence of $O(n^3)$ operations.

There is a line of research with the goal to alleviate this burden by using approximate covariance functions $\tilde{k}$ instead of k. A review is given by Candela and Rasmussen[1]. One basic idea in those approximations is to work with a set of m inducing inputs u with a reduced computational load of $O(nm^2)$. In the following, we will provide a rough idea of the FITC approximation used in the toolbox. Let K denote the $n \times n$ covariance matrix between the training points x, K_u the $m \times n$ covariance matrix between the n training points and the m inducing points, and K_{uu} the $m \times m$ covariance matrix between the m inducing points. The FITC approximation to the covariance is given by

$$K \approx \tilde{K} = Q + G,\ G = \text{diag}(g),\ g = \text{diag}(K - Q),\ Q = K_u^\top Q_{uu}^{-1} K_u,\ Q_{uu} = K_{uu} + \sigma_{n_u}^2 I,$$

where σ_{n_u} is the noise from the inducing inputs. Note that $\tilde{K}$ and K have the same diagonal elements $\text{diag}(\tilde{K}) = \text{diag}(K)$; all off-diagonal elements are the same as for Q. Internally, the necessary covariance evaluations are performed by a meta covariance function `cov/apxSparse.m`. The toolbox offers FITC versions for regression with Gaussian likelihood `inf/infGaussLik.m`, as well as for Laplace's approximation `inf/infLaplace.m`.

The user can decide whether to treat the inducing inputs u as fixed or as hyperparameters. The latter allows to adjust the inducing inputs u w.r.t. the marginal likelihood. As detailed in the documentation of `inf/apx.m`, u is treated as fixed if it is passed as the 2nd parameter of `apxSparse(cov,xu,..)`. If the hyperparameter structure `hyp` contains a field `hyp.xu` in inference method calls such as `infGaussLik(hyp,..)` or inference/prediction calls like `gp(hyp,@infGaussLik,..)` the inducing inputs u are treated as hyperparameters and can be optimised. See `doc/demoSparse.m` for an illustration.

3.8 Grid-Based Covariance Approximations

Another way to bring down computational costs is to take advantage of grid structure x. For example, in geostatistics or image processing, the training data $x \in \mathbb{R}^{n \times D}$ could be a complete 2d lattice of size $n_1 \times n_2$ as given by the axes $g_1 \in \mathbb{R}^{n_1}$, $g_2 \in \mathbb{R}^{n_2}$ so that $n = N = n_1 \cdot n_2$, $D = 2$ and $x = [\text{vec}(g_1 1^\top), \text{vec}(1 g_2^\top)]$. In general, a p-dimensional grid $U \in \mathbb{R}^{N \times D}$ is specified by a set of axis matrices $\{g_i \in \mathbb{R}^{n_i \times D_i}\}_{i=1..p}$ so that $N = \prod_{i=1}^p n_i$ and $D = \sum_{i=1}^p D_i$ where the axes do not need to be 1d nor do their components need to be sorted. As a consequence, U represents a Cartesian product of its axes $U = g_1 \times g_2 \times .. \times g_p$. The `cov/apxGrid.m` covariance function represents a Kronecker product covariance matrix

$$K_{U,U} = K_p \otimes .. \otimes K_2 \otimes K_1$$

[1] A Unifying View of Sparse Approximate Gaussian Process Regression, JMLR, 2005

whose factorisation structure is given by the grid x_g. The gain in computationial efficiency is due to the fact that matrix-vector product, determinant, inverse and eigenvalue computations decompose so that many operations with an overall cost of $\mathcal{O}(N^3)$ now only cost $\mathcal{O}(\sum_{i=1}^{p} n_i^3)$.

For off-grid data points, we can still take advantage of the computational properties of a grid-based covariance matrix $K_{U,U}$ via the structured kernel interpolation (SKI) framework aka KISS-GP by Wilson and Nickisch[2] with extensions[3]. Here, the $n \times n$ covariance K is obtained from the $N \times N$ grid covariance $K_{U,U}$ by interpolation $K \approx W_X K_{U,U} W_X^\top$, where $K_{U,U}$ is a covariance matrix formed by evaluating the user-specified kernel over a set of latent inducing inputs U, with locations that have been chosen to create algebraic structure in $K_{U,U}$ that we can exploit for efficiency. Here, the interpolation matrix $W_X \in \mathbb{R}^{n \times N}$ is extremely sparse; i.e., for local cubic interpolation W_X contains only 4^D nonzeros per row, where D is the diata dimension. In addition W_X is row-normalised $1_n = W_X 1_N$. The structure in $K_{U,U}$ alongside the sparsity of W_X, allows for very fast MVMs with the SKI approximate covariance matrix K over the inputs x enabling fast inference and prediction.

Internally, we use a meta covariance function `cov/apxGrid.m` to represent the Kronecker covariance matrix and a Gaussian regression inference method `inf/infGaussLik.m`. We also support incomplete grids where $n < N$. A good starting point is Yunus Saatçi's PhD thesis[4]. For incomplete grids, we use the interpolation-based extensions by Wilson et al.[5] where conjugate gradients and a determinant approximations are used. See `doc/demoGrid1d.m` and `doc/demoGrid2d.m` for an illustration. We also offer non-Gaussian likelihoods as described by Seth Flaxman[6] so that `inf/infLaplace.m` can be used.

3.9 State Space Representation of GPs

GP models with covariance functions with a Markovian structure can be transformed into equivalent discrete state space models where inference can be done in linear time $\mathcal{O}(n)$. Exact models can be derived for sum, product, linear, noise, constant, Matérn (half-integer), Ornstein–Uhlenbeck, and Wiener covariance functions. Other common covariance functions can be approximated by their Markovian counterparts, including squared exponential, rational quadratic, and periodic covariance functions.

A state space model describes the evolution of a dynamical system at different time instances t_i, $i = 1, 2, \dots$ by

$$f_i \sim \mathbb{P}(f_i | f_{i-1}), \quad y_i \sim \mathbb{P}(y_i | f_i),$$

where $f_i := f(t_i) \in \mathbb{R}^d$ and $f_0 \sim \mathbb{P}(f_0)$ with f_i being the latent (hidden/unobserved) variable and y_i being the observed variable. In continuous time, a simple dynamical system able to represent many covariance functions is given by the following linear time-invariant stochastic differential equation:

$$\dot{f}(t) = F\,f(t) + L\,w(t), \quad y_i = H\,f(t_i) + \epsilon_i,$$

where $w(t)$ is an s-dimensional white noise process, the measurement noise $\epsilon_i \sim \mathcal{N}(0, \sigma_n^2)$ is Gaussian, and $F \in \mathbb{R}^{d \times d}$, $L \in \mathbb{R}^{d \times s}$, $H \in \mathbb{R}^{1 \times d}$ are the feedback, noise effect, and measurement matrices, respectively. The initial state is distributed according to $f_0 \sim \mathcal{N}(0, P_0)$.

The latent GP is recovered by $f(t) = Hf(t)$ and $w(t) \in \mathbb{R}^s$ is a multivariate white noise process with spectral density matrix $Q_c \in \mathbb{R}^{s \times s}$. For discrete values, this translates into

$$f_i \sim \mathcal{N}(A_{i-1} f_{i-1}, Q_{i-1}), \quad y_i \sim \mathbb{P}(y_i | H\,L f_i),$$

[2] Kernel Interpolation for Scalable Structured Gaussian Processes, ICML, 2015
[3] Thoughts on Massively Scalable Gaussian Processes, TR, 2015.
[4] Scalable Inference for Structured Gaussian Process Models, University of Cambridge, 2011
[5] Fast Kernel Learning for Multidimensional Pattern Extrapolation, NIPS, 2014
[6] Fast Kronecker inference in Gaussian processes with non-Gaussian likelihoods, ICML, 2015

Algorithm 1 Kalman (forward) filtering.

```
Input: {t_i} , y                                   # training inputs and targets
       {A_i}, {Q_i}, H, P_0                        # state space model
       W,b                                         # likelihood eff. precision and location
for i = 1 to n do
  if i == 1 then
    m_i ← 0;  P_i ← P_0                            # init
  else
    m_i ← A_i m_{i−1}; P_i ← A_i P_{i−1} A_i^⊤ + Q_i    # predict
  end if
  if has label y_i then
    μ_f ← H m_i;  u ← P_i H^⊤;  σ_f^2 ← H u        # latent
    z_i ← W_ii σ_f^2 + 1
    k_i ← W_ii u/z_i;  P_i ← P_i − k_i u^⊤         # variance
    c_i ← W_ii μ_f − b_i;  m_i ← m_i − u c_i/z_i   # mean
  end if
end for
log det(I + W^{1/2} K W^{1/2}) ← Σ_i log z_i
```

Algorithm 2 Rauch–Tung–Striebel (backward) smoothing.

```
Input: {m_i}, {P_i}                                # Kalman filter output
       {A_i}, {Q_i}                                # state space model
for i = n down to 2 do
  m ← A_i m_{i−1};  P ← A_i P_{i−1} A_i^⊤ + Q_i    # predict
  G_i ← P_{i−1} A_i^⊤ P^{−1};  Δm_{i−1} ← G_i(m_i − m)
  P_{i−1} ← P_{i−1} + G_i(P_i − P) G_i^⊤            # variance
  m_{i−1} ← m_{i−1} + Δm_{i−1}                      # mean
end for
```

with $f_0 \sim \mathcal{N}(0, P_0)$. The discrete-time matrices are

$$A_i = A[\Delta t_i] = e^{\Delta t_i F},$$
$$Q_i = \int_0^{\Delta t_i} e^{(\Delta t_k - \tau)F} L\, Q_c\, L^\top e^{(\Delta t_i - \tau)F^\top} d\tau,$$

where $\Delta t_i = t_{i+1} - t_i \geqslant 0$.

For stationary covariances $k(t, t') = k(t - t')$, the stationary state is distributed by $f_\infty \sim \mathcal{N}(0, P_\infty)$ and the stationary covariance can be found by solving the Lyapunov equation

$$\dot{P}_\infty = F\, P_\infty + P_\infty\, F^\top + L\, Q_c\, L^\top = 0,$$

which leads to the identity $Q_i = P_\infty - A_i\, P_\infty\, A_i^\top$.

In practice, the evaluation of the n discrete-time transition matrices $A_i = e^{\Delta t_i F}$ and the noise covariance matrices Q_i (in the stationary case) for different values of Δt_i is a computational challenge. Since the matrix exponential $\psi : s \mapsto e^{sX}$ is smooth, its evaluation can be accurately approximated by convolution interpolation.

From the discrete set of matrices, all the necessary computations can be done using Kalman filtering and Kalman smoothing as detailed in Algorithms 1 and 2.

Internally, we use a meta covariance function `cov/apxState.m` to represent the state space represen-

tation. A good starting point is Arno Solin's PhD thesis[7]. See `doc/demoState.m` for an illustration. We also offer non-Gaussian likelihoods [8] so that `inf/infLaplace.m` and `inf/infVB.m` can be used. EP is not fully functional; we offer single-sweep EP aka assumed density filtering (ADF).

[7] Stochastic Differential Equation Methods for Spatio-Temporal Gaussian Process Regression, Aalto University, 2016

[8] State Space Gaussian Processes with Non-Gaussian Likelihood, ICML, 2018

4 Likelihood Functions

A likelihood function $p_\rho(y|f)$ (with hyperparameters ρ) is a conditional density $\int p_\rho(y|f)dy = 1$ defined for scalar latent function values f and outputs y. In the GPML toolbox, we use iid. likelihoods $p_\rho(y|f) = \prod_{i=1}^n p_\rho(y_i|f_i)$. The approximate inference engine does not explicitly distinguish between classification and regression likelihoods: it is fully generic in the likelihood allowing to use a single code in the inference step.

Likelihood functionality is needed both during inference and while predicting.

4.1 Prediction

A prediction at x_* conditioned on the data $\mathcal{D} = (X, y)$ (as implemented in `gp.m`) consists of the predictive mean μ_{y_*} and variance $\sigma^2_{y_*}$ which are computed from the the latent marginal moments μ_{f_*}, $\sigma^2_{f_*}$ i.e. the Gaussian marginal approximation $\mathcal{N}(f_*|\mu_{f_*}, \sigma^2_{f_*})$ via

$$p(y_*|\mathcal{D}, x_*) = \int p(y_*|f_*)p(f_*|\mathcal{D}, x_*)df_* \approx \int p(y_*|f_*)\mathcal{N}(f_*|\mu_{f_*}, \sigma^2_{f_*})df_*. \quad (3)$$

The moments are given by $\mu_{y_*} = \int y_* p(y_*|\mathcal{D}, x_*)dy_*$ and $\sigma^2_{y_*} = \int (y_* - \mu_{y_*})^2 p(y_*|\mathcal{D}, x_*)dy_*$. The likelihood call

- `[lp,ymu,ys2] = lik(hyp, [], fmu, fs2)`

does exactly this. Evaluation of the logarithm of $p_{y_*} = p(y_*|\mathcal{D}, x_*)$ for values y_* can be done via

- `[lp,ymu,ys2] = lik(hyp, y, fmu, fs2)`

where `lp` contains the number $\ln p_{y_*}$.

Using the moments of the likelihood $\mu(f_*) = \int y_* p(y_*|f_*)dy_*$ and $\sigma^2(f_*) = \int (y_* - \mu(f_*))^2 p(y_*|f_*)dy_*$ we obtain for the predictive moments the following (exact) expressions

$$\begin{aligned}
\mu_{y_*} &= \int \mu(f_*)p(f_*|\mathcal{D}, x_*)df_*, \text{ and} \\
\sigma^2_{y_*} &= \int \left[\sigma^2(f_*) + (\mu(f_*) - \mu_{y_*})^2\right] p(f_*|\mathcal{D}, x_*)df_*.
\end{aligned}$$

1. The binary case is simple since $y_* \in \{-1, +1\}$ and $1 = p_{y_*} + p_{-y_*}$. Using $\pi_* = p_{+1}$, we find

$$\begin{aligned}
p_{y_*} &= \begin{cases} \pi_* & y_* = +1 \\ 1 - \pi_* & y_* = -1 \end{cases} \\
\mu_{y_*} &= \sum_{y_*=\pm 1} y_* p(y_*|\mathcal{D}, x_*) = 2 \cdot \pi_* - 1 \in [-1, 1], \quad \text{and} \\
\sigma^2_{y_*} &= \sum_{y_*=\pm 1} (y_* - \mu_{y_*})^2 p(y_*|\mathcal{D}, x_*) = 4 \cdot \pi_*(1 - \pi_*) \in [0, 1].
\end{aligned}$$

2. The continuous case for homoscedastic likelihoods depending on $r_* = y_* - f_*$ only and having noise variance $\sigma^2(f_*) = \sigma^2_n$ is also simple since the identity $p(y_*|f_*) = p(y_* - f_*|0)$ allows to substitute $y_* \leftarrow y_* + f_*$ yielding $\mu(f_*) = f_* + \int y_* p(y_*|0)dy_*$ and assuming $\int y_* p(y_*|0)dy_* = 0$ we arrive at

$$\begin{aligned}
\mu_{y_*} &= \mu_{f_*}, \text{ and} \\
\sigma^2_{y_*} &= \sigma^2_{f_*} + \sigma^2_n.
\end{aligned}$$

3. The generalised linear model (GLM) case is also feasible. Evaluation of the predictive distribution is done by quadrature

$$p_{y_*} = \int p(y_*|f_*)p(f_*|\mathcal{D}, x_*)df_* \approx \int p(y_*|f_*)\mathcal{N}(f_*|\mu_{f_*}, \sigma^2_{f_*})df_*.$$

   For GLMs the mean is given by $\mu(f_*) = g(f_*)$ and the variance is usually given by a simple function of the mean $\sigma^2(f_*) = v(g(f_*))$, hence we use Gaussian-Hermite quadrature with $\mathcal{N}(f_*|\mu_{f_*}, \sigma^2_{f_*}) \approx p(f_*|\mathcal{D}, x_*)$ to compute

$$\mu_{y_*} = \int g(f_*)p(f_*|\mathcal{D}, x_*)df_*, \text{ and}$$
$$\sigma^2_{y_*} = \int \left[v(g(f_*)) + (g(f_*) - \mu_{y_*})^2\right] p(f_*|\mathcal{D}, x_*)df_* \neq v(\mu_{y_*}).$$

4. Finally the warped Gaussian likelihood predicitive distribution with strictly monotonically increasing warping function g is given by the expression

$$p(y_*|\mathcal{D}, x_*) = g'(y_*)\mathcal{N}\left(g(y_*)|\mu_{f_*}, \sigma^2_n + \sigma^2_{f_*}\right)$$

   so that the predictive moments can be computed by Gaussian-Hermite quadrature.

In the following, we will detail how and which likelihood functions are implemented in the GPML toolbox. Further, we will mention dependencies between likelihoods and inference methods and provide some analytical expressions in addition to some likelihood implementations.

4.2 Interface

The likelihoods are in fact the most challenging object in our implementation. Different inference algorithms require different aspects of the likelihood to be computed, therefore the interface is rather involved as detailed below.

20 ⟨likFunctions.m 20⟩≡

```
% likelihood functions are provided to be used by the gp.m function:
%
%   likErf         (Error function, classification, probit regression)
%   likLogistic    (Logistic,       classification, logit  regression)
%   likUni         (Uniform likelihood, classification)
%
%   likGauss       (Gaussian, regression)
%   likGaussWarp   (Warped Gaussian, regression)
%   likGumbel      (Gumbel likelihood for extremal values)
%   likLaplace     (Laplacian or double exponential, regression)
%   likSech2       (Sech-square, regression)
%   likT           (Student's t, regression)
%
%   likPoisson     (Poisson regression, count data)
%   likNegBinom    (Negativ binomial regression, count data)
%   likGamma       (Nonnegative regression, positive data)
%   likExp         (Nonnegative regression, positive data)
%   likInvGauss    (Nonnegative regression, positive data)
%   likLogGauss    (Nonnegative regression, positive data)
%   likBeta        (Beta regression, interval data)
%
%   likMix         (Mixture of individual likelihood functions)
```

```
%
% The likelihood functions have three possible modes, the mode being selected
% as follows (where "lik" stands for any likelihood function in "lik/lik*.m".):
%
% 1) With one or no input arguments:          [REPORT NUMBER OF HYPERPARAMETERS]
%
%    s = lik OR s = lik(hyp)
%
% The likelihood function returns a string telling how many hyperparameters it
% expects, using the convention that "D" is the dimension of the input space.
% For example, calling "likLogistic" returns the string '0'.
%
%
% 2) With three or four input arguments:                       [PREDICTION MODE]
%
%    lp = lik(hyp, y, mu) OR [lp, ymu, ys2] = lik(hyp, y, mu, s2)
%
% This allows to evaluate the predictive distribution. Let p(y_*|f_*) be the
% likelihood of a test point and N(f_*|mu,s2) an approximation to the posterior
% marginal p(f_*|x_*,x,y) as returned by an inference method. The predictive
% distribution p(y_*|x_*,x,y) is approximated by.
%   q(y_*) = \int N(f_*|mu,s2) p(y_*|f_*) df_*
%
%   lp = log( q(y) ) for a particular value of y, if s2 is [] or 0, this
%                    corresponds to log( p(y|mu) )
%   ymu and ys2      the mean and variance of the predictive marginal q(y)
%                    note that these two numbers do not depend on a particular
%                    value of y
%  All vectors have the same size.
%
%
% 3) With five or six input arguments, the fifth being a string [INFERENCE MODE]
%
% [varargout] = lik(hyp, y, mu, s2, inf) OR
% [varargout] = lik(hyp, y, mu, s2, inf, i)
%
% There are three cases for inf, namely a) infLaplace, b) infEP and c) infVB.
% The last input i, refers to derivatives w.r.t. the ith hyperparameter.
%
% a1) [lp,dlp,d2lp,d3lp] = lik(hyp, y, f, [], 'infLaplace')
% lp, dlp, d2lp and d3lp correspond to derivatives of the log likelihood
% log(p(y|f)) w.r.t. to the latent location f.
%   lp = log( p(y|f) )
%  dlp = d   log( p(y|f) ) / df
% d2lp = d^2 log( p(y|f) ) / df^2
% d3lp = d^3 log( p(y|f) ) / df^3
%
% a2) [lp_dhyp,dlp_dhyp,d2lp_dhyp] = lik(hyp, y, f, [], 'infLaplace', i)
% returns derivatives w.r.t. to the ith hyperparameter
%   lp_dhyp = d   log( p(y|f) ) / (     dhyp_i)
%  dlp_dhyp = d^2 log( p(y|f) ) / (df   dhyp_i)
% d2lp_dhyp = d^3 log( p(y|f) ) / (df^2 dhyp_i)
%
%
% b1) [lZ,dlZ,d2lZ] = lik(hyp, y, mu, s2, 'infEP')
% let Z = \int p(y|f) N(f|mu,s2) df then
%   lZ =     log(Z)
%  dlZ = d   log(Z) / dmu
```

```
% d2lZ = d^2 log(Z) / dmu^2
%
% b2) [dlZhyp] = lik(hyp, y, mu, s2, 'infEP', i)
% returns derivatives w.r.t. to the ith hyperparameter
% dlZhyp = d log(Z) / dhyp_i
%
%
% c1) [b,z] = lik(hyp, y, [], ga, 'infVB')
% ga is the variance of a Gaussian lower bound to the likelihood p(y|f).
%   p(y|f) \ge exp( b*(f+z) - (f+z).^2/(2*ga) - h(ga)/2 ) \propto N(f|b*ga-z,ga)
% The function returns the linear part b and z.
%
% Cumulative likelihoods are designed for binary classification. Therefore, they
% only look at the sign of the targets y; zero values are treated as +1.
%
% Some examples for valid likelihood functions:
%      lik = @likLogistic;
%      lik = {'likMix',{'likUni',@likErf}}
%      lik = {@likPoisson,'logistic'};
%
% See the help for the individual likelihood for the computations specific to
% each likelihood function.
%
⟨gpml copyright 6a⟩
```

4.3 Implemented Likelihood Functions

The following table enumerates all (currently) implemented likelihood functions that can be found at `lik/lik<NAME>.m` and their respective set of hyperparameters $\boldsymbol{\rho}$.

lik<NAME>	regression $y_i \in \mathbb{R}$	$p_\rho(y_i \mid f_i) =$	$\boldsymbol{\rho} =$
Gauss	Gaussian	$\mathcal{N}(y_i \mid f_i, \sigma^2) = \frac{1}{\sqrt{2\pi}\sigma}\exp\left(-\frac{(y_i-f_i)^2}{2\sigma^2}\right)$	$\{\ln\sigma\}$
GaussWarp	Warped Gaussian	$\mathcal{N}(g_\theta(y_i) \mid f_i, \sigma^2) g'_\theta(y_i)$	$\{\theta_1, .., \theta_{n_g}, \ln\sigma\}$
Gumbel	Gumbel	$\frac{\pi}{\sigma\sqrt{6}}\exp(-z_i - e^{-z_i})$, $z_i = \gamma + \frac{s\cdot\pi(y_i-f_i)}{\sigma\sqrt{6}}$, $\lvert s\rvert = 1$	$\{\ln\sigma\}$
Sech2	Sech-squared	$\frac{\tau}{2\cosh^2(\tau(y_i-f_i))}$, $\tau = \frac{\pi}{2\sigma\sqrt{3}}$	$\{\ln\sigma\}$
Laplace	Laplacian	$\frac{1}{2b}\exp\left(-\frac{\lvert y_i-f_i\rvert}{b}\right)$, $b = \frac{\sigma}{\sqrt{2}}$	$\{\ln\sigma\}$
T	Student's t	$\frac{\Gamma(\frac{\nu+1}{2})}{\Gamma(\frac{\nu}{2})}\frac{1}{\sqrt{\nu\pi}\sigma}\left(1+\frac{(y_i-f_i)^2}{\nu\sigma^2}\right)^{-\frac{\nu+1}{2}}$	$\{\ln(\nu-1), \ln\sigma\}$
lik<NAME>	classification $y_i \in \{\pm 1\}$	$p_\rho(y_i \mid f_i) =$	$\boldsymbol{\rho} =$
Erf	Error function	$\int_{-\infty}^{y_i f_i}\mathcal{N}(t)dt$	$\emptyset$
Logistic	Logistic function	$\frac{1}{1+\exp(-y_i f_i)}$	$\emptyset$
Uni	Label noise	$\frac{1}{2}$	$\emptyset$
lik<NAME>	count data $y_i \in \mathbb{N}$	$p_\rho(y_i \mid f_i) =$ where $\mu = g(f_i)$	$\boldsymbol{\rho} =$
Poisson	Poisson	$\mu^{y_i}e^{-\mu}/y_i!$	$\emptyset$
NegBinom	Negative Binomial	$\binom{y_i+r-1}{y_i} r^r \mu^{y_i}/(r+\mu)^{r+y_i}$	$\{\ln r\}$
lik<NAME>	nonnegative data $y_i \in \mathbb{R}_+ \backslash \{0\}$	$p_\rho(y_i \mid f_i) =$ where $\mu = g(f_i)$	$\boldsymbol{\rho} =$
Weibull	Weibull, $\gamma_1 = \Gamma(1+1/\kappa)$	$\kappa\gamma_1/\mu\,(y_i\gamma_1/\mu)^{\kappa-1}\exp(-(y_i\gamma_1/\mu)^\kappa)$	$\{\ln\kappa\}$
Gamma	Gamma	$\frac{\alpha^\alpha y_i^{\alpha-1}}{\Gamma(\alpha)}\mu^{-\alpha}\exp\left(-\frac{y_i\alpha}{\mu}\right)$	$\{\ln\alpha\}$
Exp	Exponential	$\mu^{-1}\exp\left(-\frac{y_i}{\mu}\right)$	$\emptyset$
InvGauss	Inverse Gaussian	$\sqrt{\frac{\lambda}{2\pi y_i^3}}\exp\left(-\frac{\lambda(y_i-\mu)^2}{2\mu^2 y_i}\right)$	$\{\ln\lambda\}$
lik<NAME>	interval data $y_i \in [0,1]$	$p_\rho(y_i \mid f_i) =$ where $\mu = g(f_i)$	$\boldsymbol{\rho} =$
Beta	Beta	$\frac{\Gamma(\phi)}{\Gamma(\mu\phi)\Gamma((1-\mu)\phi)} y_i^{\mu\phi-1}(1-y_i)^{(1-\mu)\phi-1}$	$\{\ln\phi\}$
Composite likelihood functions $[p_1(y_i \mid f_i), p_1(y_i \mid f_i), ..] \mapsto p_\rho(y_i \mid f_i)$			
Mix	Mixture	$\sum_j \alpha_j p_j(y_i \mid f_i)$	$\{\ln\alpha_1, \ln\alpha_2, ..\}$

4.4 Usage of Implemented Likelihood Functions

Some code examples taken from `doc/usageLik.m` illustrate how to use simple and composite likelihood functions to specify a GP model.

Syntactically, a likelihood function `lf` is defined by

```
lk := 'func' | @func // simple

lf := {lk} | {param, lk} | {lk, {lk, .., lk}} // composite
```

i.e., it is either a string containing the name of a likelihood function, a pointer to a likelihood function or one of the former in combination with a cell array of likelihood functions and an additional list of parameters.

23 ⟨doc/usageLik.m 23⟩≡

```
% demonstrate usage of likelihood functions
%
% See also likFunctions.m.
%
⟨gpml copyright 6a⟩
clear all, close all
n = 5; f = randn(n,1);        % create random latent function values

```

```
% set up simple classification likelihood functions
yc = sign(f);
lc0 = {'likErf'};     hypc0 = [];   % no hyperparameters are needed
lc1 = {@likLogistic}; hypc1 = [];    % also function handles are OK
lc2 = {'likUni'};     hypc2 = [];
lc3 = {'likMix',{'likUni',@likErf}}; hypc3 = log([1;2]); %mixture

% set up simple regression likelihood functions
yr = f + randn(n,1)/20;
sn = 0.1;                                % noise standard deviation
lr0 = {'likGauss'};   hypr0 = log(sn);
lr1 = {'likLaplace'}; hypr1 = log(sn);
lr2 = {'likSech2'};   hypr2 = log(sn);
nu = 4;                              % number of degrees of freedom
lr3 = {'likT'};       hypr3 = [log(nu-1); log(sn)];
lr4 = {'likMix',{lr0,lr1}}; hypr4 = [log([1,2]);hypr0;hypr1];

a = 1; % set up warped Gaussian with g(y) = y + a*sign(y).*y.^2
lr5 = {'likGaussWarp',['poly2']}; hypr5 = log([a;sn]);
lr6 = {'likGumbel','+'}; hypr6 = log(sn);

% set up Poisson/negative binomial regression
yp = fix(abs(f)) + 1;
lp0 = {@likPoisson,'logistic'};        hypp0 = [];
lp1 = {@likPoisson,{'logistic2',0.1}}; hypp1 = [];
lp2 = {@likPoisson,'exp'};             hypp2 = [];
ln1 = {@likNegBinom,'logistic2'};      hypn1 = [];

% set up other GLM likelihoods for positive or interval regression
lg1 = {@likGamma,'logistic'};            al = 2;    hyp.lik = log(al);
lg2 = {@likInvGauss,'exp'};              lam = 1.1; hyp.lik = log(lam);
lg3 = {@likBeta,'expexp'};               phi = 2.1; hyp.lik = log(phi);
lg4 = {@likBeta,'logit'};                phi = 4.7; hyp.lik = log(phi);
lg5 = {@likWeibull,{'logistic2',0.01}};  ka = 0.5;  hyp.lik = log(ka);

% 0) specify the likelihood function
lik = lc0; hyp = hypc0; y = yc;
% lik = lr4; hyp = hypr4; y = yr;
% lik = lp1; hyp = hypp1; y = yp;

% 1) query the number of parameters
feval(lik{:})

% 2) evaluate the likelihood function on f
exp(feval(lik{:},hyp,y,f))

% 3a) evaluate derivatives of the likelihood
[lp,dlp,d2lp,d3lp] = feval(lik{:}, hyp, y, f, [], 'infLaplace');

% 3b) compute Gaussian integrals w.r.t. likelihood
mu = f; s2 = rand(n,1);
[lZ,dlZ,d2lZ] = feval(lik{:}, hyp, y, mu, s2, 'infEP');

% 3c) obtain lower bound on likelihood
ga = rand(n,1);
[b,z] = feval(lik{:}, hyp, y, [], ga, 'infVB');
```

4.5 Compatibility Between Likelihoods and Inference Methods

The following table lists all possible combinations of likelihood function and inference methods.

Likelihood \ Inference	Gaussian Likelihood	Laplace	VB	EP	KL	MCMC	LOO	Type, Output Domain	Alternative Names
	approx. cov. possible, Table3.6			no approx. cov. possible, Table3.6					
Gaussian	✓	✓	✓	✓	✓	✓	✓	regression, $\mathbb{R}$	
Warped Gaussian		✓	✓	✓	✓	✓	✓	regression, $\mathbb{R}$	
Gumbel		✓			✓	✓	✓	regression, $\mathbb{R}$	
Sech-squared		✓	✓	✓	✓	✓	✓	regression, $\mathbb{R}$	logistic distribution
Laplacian		✓	✓	✓	✓	✓	✓	regression, $\mathbb{R}$	double exponential
Student's t		✓	✓		✓	✓	✓	regression, $\mathbb{R}$	
Mixture		✓		✓	✓	✓	✓		mixing meta likelihood
Error function		✓		✓	✓	✓	✓	classification, $\{\pm 1\}$	probit regression
Logistic function		✓	✓	✓	✓	✓	✓	classification, $\{\pm 1\}$	logit regression
Uniform		✓	✓	✓	✓	✓	✓	classification, $\{\pm 1\}$	label noise
Weibull		✓				✓	✓	positive data, $\mathbb{R}_+ \backslash \{0\}$	nonnegative regression
Gamma		✓				✓	✓	positive data, $\mathbb{R}_+ \backslash \{0\}$	nonnegative regression
Exp		✓				✓	✓	positive data, $\mathbb{R}_+ \backslash \{0\}$	nonnegative regression
Inverse Gaussian		✓				✓	✓	positive data, $\mathbb{R}_+ \backslash \{0\}$	nonnegative regression
Poisson		✓		(✓)*	✓	✓	✓	count data, $\mathbb{N}$	Poisson regression
Negative Binomial		✓				✓	✓	count data, $\mathbb{N}$	negative binomial regression
Beta		✓				✓	✓	interval data, $[0, 1]$	beta regression

(✓)* EP might not converge in some cases since quadrature is used.

Exact inference is only tractable for Gaussian likelihoods. Expectation propagation together with Student's t likelihood is inherently unstable due to non-log-concavity. Laplace's approximation for Laplace likelihoods is not sensible because at the mode the curvature and the gradient is undefined due to the non-differentiable peak of the Laplace distribution. Special care has been taken for the non-convex optimisation problem imposed by the combination Student's t likelihood and Laplace's approximation.

4.6 Gaussian Likelihood

The Gaussian likelihood is the simplest likelihood because the posterior distribution is not only Gaussian but can be computed analytically. In principle, the Gaussian likelihood would only be operated in conjunction with the exact inference method but we chose to provide compatibility with all other inference algorithms as well because it enables code testing and allows to switch between different regression likelihoods very easily.

25 ⟨lik/likGauss.m 25⟩≡

```
function [varargout] = likGauss(hyp, y, mu, s2, inf, i)

% likGauss - Gaussian likelihood function for regression. The expression for the
% likelihood is
%   likGauss(t) = exp(-(t-y)^2/2*sn^2) / sqrt(2*pi*sn^2),
% where y is the mean and sn is the standard deviation.
%
% The hyperparameters are:
%
% hyp = [  log(sn)  ]
%
% Several modes are provided, for computing likelihoods, derivatives and moments
% respectively, see likFunctions.m for the details. In general, care is taken
% to avoid numerical issues when the arguments are extreme.
%
⟨gpml copyright 6a⟩
%
```

```
% See also likFunctions.m.

if nargin<3, varargout = {'1'}; return; end   % report number of hyperparameters

sn2 = exp(2*hyp);

if nargin<5                                 % prediction mode if inf is not present
  ⟨Prediction with Gaussian likelihood 26a⟩
else
  switch inf
  case 'infLaplace'
    ⟨Laplace's method with Gaussian likelihood 26b⟩
  case 'infEP'
    ⟨EP inference with Gaussian likelihood 27a⟩
  case 'infVB'
    ⟨Variational Bayes inference with Gaussian likelihood 27b⟩
  end
end
```

26a ⟨Prediction with Gaussian likelihood 26a⟩≡ (25)

```
if isempty(y),  y = zeros(size(mu)); end
s2zero = 1; if nargin>3&&numel(s2)>0&&norm(s2)>eps, s2zero = 0; end  % s2==0 ?
if s2zero                                                  % log probability
  lp = -(y-mu).^2./sn2/2-log(2*pi*sn2)/2; s2 = 0;
else
  lp = likGauss(hyp, y, mu, s2, 'infEP');                          % prediction
end
ymu = {}; ys2 = {};
if nargout>1
  ymu = mu;                                                 % first y moment
  if nargout>2
    ys2 = s2 + sn2;                                        % second y moment
  end
end
varargout = {lp,ymu,ys2};
```

The Gaussian likelihood function has a single hyperparameter ρ, the log of the noise standard deviation σ_n.

4.6.1 Exact Inference

Exact inference doesn't require any specific likelihood related code; all computations are done directly by the inference method, section 3.1.

4.6.2 Laplace's Approximation

26b ⟨Laplace's method with Gaussian likelihood 26b⟩≡ (25)

```
if nargin<6                                            % no derivative mode
  if isempty(y), y=0; end
  ymmu = y-mu; dlp = {}; d2lp = {}; d3lp = {};
  lp = -ymmu.^2/(2*sn2) - log(2*pi*sn2)/2;
  if nargout>1
    dlp = ymmu/sn2;                      % dlp, derivative of log likelihood
    if nargout>2                    % d2lp, 2nd derivative of log likelihood
      d2lp = -ones(size(ymmu))/sn2;
      if nargout>3                  % d3lp, 3rd derivative of log likelihood
        d3lp = zeros(size(ymmu));
```

```
      end
    end
  end
  varargout = {lp,dlp,d2lp,d3lp};
else                                                          % derivative mode
  lp_dhyp = (y-mu).^2/sn2 - 1;  % derivative of log likelihood w.r.t. hypers
  dlp_dhyp = 2*(mu-y)/sn2;                                   % first derivative,
  d2lp_dhyp = 2*ones(size(mu))/sn2;   % and also of the second mu derivative
  varargout = {lp_dhyp,dlp_dhyp,d2lp_dhyp};
end
```

4.6.3 Expectation Propagation

27a ⟨EP inference with Gaussian likelihood 27a⟩≡ (25)

```
if nargin<6                                                  % no derivative mode
  lZ = -(y-mu).^2./(sn2+s2)/2 - log(2*pi*(sn2+s2))/2;     % log part function
  dlZ = {}; d2lZ = {};
  if nargout>1
    dlZ  = (y-mu)./(sn2+s2);                        % 1st derivative w.r.t. mean
    if nargout>2
      d2lZ = -1./(sn2+s2);                          % 2nd derivative w.r.t. mean
    end
  end
  varargout = {lZ,dlZ,d2lZ};
else                                                          % derivative mode
  dlZhyp = ((y-mu).^2./(sn2+s2)-1) ./ (1+s2./sn2);   % deriv. w.r.t. hyp.lik
  varargout = {dlZhyp};
end
```

4.6.4 Variational Bayes

27b ⟨Variational Bayes inference with Gaussian likelihood 27b⟩≡ (25)

```
% variational lower site bound
% t(s) = exp(-(y-s)^2/2sn2)/sqrt(2*pi*sn2)
% the bound has the form: (b+z/ga)*f - f.^2/(2*ga) - h(ga)/2
n = numel(s2); b = zeros(n,1); y = y.*ones(n,1); z = y;
varargout = {b,z};
```

4.7 Warped Gaussian Likelihood

Starting from the likelihood $p(y|f)$ we are sometimes facing the situation where the data $y \in \mathcal{Y} \subseteq \mathbb{R}$ is not distributed according to $p(y|f)$ but some nonlinear transformation of the data $g(y) = z$ so that $z \sim p(z|f)$. Here, the warping function $g : \mathcal{Y} \to \mathbb{R}$ needs to be strictly monotonically increasing i.e. $g'(y) > 0$. Formally, we start from the fact that $p(z|f)$ integrates to one and use the derivative $dz = g'(y)dy$ to substitute

$$\int p(z|f)dz = 1 = \int p_g(y|f)dy, \; p_g(y|f) = p(g(y)|f)g'(y)$$

where we have defined the log warped likelihood $\ln p_g(y|f) = \ln p(g(y)|f) + \ln g'(y)$. The interesting bit is that approximate inference methods such as `infExact`, `infLaplace`, `infEP`, `infVB`, `infKL` remain fully feasible; only prediction and derivatives become more involved. The usual GP inference is recovered by using the identity warping function $g : y \mapsto y$. The construction works in princple for any likelihood but our implementation in `likGaussWarp` is limited to the Gaussian likelihood.

Hyperparameter derivatives

Hyperparameter derivatives for `infLaplace` are obtained as follows

$$\begin{aligned}
\frac{\partial}{\partial\theta}\ln\frac{\partial^k}{\partial f^k}p_g(y|f) &= \frac{\partial}{\partial\theta}\ln\frac{\partial^k}{\partial f}p(g(y)|f) + \frac{\partial}{\partial\theta}\frac{\partial^k}{\partial f^k}\ln g'(y),\ k=0,1,2 \\
&= -\frac{\partial^{k+1}}{\partial f^{k+1}}\ln p(g(y)|f)\frac{\partial}{\partial\theta}g(y) + \frac{\partial}{\partial\theta}\frac{\partial^k}{\partial f^k}\ln g'(y).
\end{aligned}$$

Similarly for `infEP` the derivatives are given by

$$\begin{aligned}
\frac{\partial}{\partial\theta}\ln\int p_g(y|f)\mathcal{N}(f|\mu,\sigma^2)df &= \frac{\partial}{\partial\theta}\ln\int p(g(y)|f)\mathcal{N}(f|\mu,\sigma^2)df + \frac{\partial}{\partial\theta}\ln g'(y) \\
&= -\frac{\partial}{\partial\mu}\ln\int p(g(y)|f)\mathcal{N}(f|\mu,\sigma^2)df\frac{\partial}{\partial\theta}g(y) + \frac{\partial}{\partial\theta}\ln g'(y).
\end{aligned}$$

This trick above works for any homoscedastic likelihood where $p(y|f) = p(y+\beta|f+\beta)$ such as `likGauss`, `likLaplace`, `likSech2` and `likT`.

Predictive moments

As detailed in 4, the predictive distribution is – for Gaussian likelihood – given by

$$\begin{aligned}
p(z_*|\mathcal{D},x_*) &= \int p(z_*|f_*)p(f_*|\mathcal{D},x_*)df_* = \int \mathcal{N}(z_*|f_*,\sigma_n^2)\mathcal{N}(f_*|\mu_{f_*},\sigma_{f_*}^2)df_* \\
&= \mathcal{N}(z_*|\mu_{f_*},\sigma_n^2+\sigma_{f_*}^2),\ \text{where } z_* = g(y_*) \\
p(y_*|\mathcal{D},x_*) &= g'(y_*)\mathcal{N}(g(y_*)|\mu_{f_*},\sigma_n^2+\sigma_{f_*}^2).
\end{aligned}$$

Hence, the predictive moments are obtained by the 1d integrals

$$\begin{aligned}
\mu_{y_*} &= \int y_* g'(y_*)\mathcal{N}(g(y_*)|\mu_{f_*},\sigma_n^2+\sigma_{f_*}^2)dy_* \\
&= \int g^{-1}(z_*)\mathcal{N}(z_*|\mu_{f_*},\sigma_n^2+\sigma_{f_*}^2)dz_*,\ \text{and} \\
\sigma_{y_*}^2 &= \int (y_*-\mu_{y_*})^2 g'(y_*)\mathcal{N}(g(y_*)|\mu_{f_*},\sigma_n^2+\sigma_{f_*}^2)dy_* \\
&= \int (g^{-1}(z_*)-\mu_{y_*})^2\mathcal{N}(z_*|\mu_{f_*},\sigma_n^2+\sigma_{f_*}^2)dz_*.
\end{aligned}$$

4.8 Gumbel Likelihood

Distributions of extrema are well captured by the Gumbel distribution

$$p(y) = \frac{1}{\beta}\exp\left(-z-e^{-z}\right),\ z = s\frac{y-\eta}{\beta},\ s\in\{\pm1\}$$

with mean $\mu = \eta+\beta\gamma$ and variance $\sigma^2 = \pi^2\beta^2/6$ where $\gamma = 0.57721566490153$ denotes Euler–Mascheroni's constant. Skewness is approximately given by $1.1395s$ where s is a sign switching between left and right skewness and kurtosis is 12/5. The final expression for the Gumbel likelihood is

$$p(y|f) = \frac{\pi}{\sigma\sqrt{6}}\exp\left(-z-e^{-z}\right),\ z = \gamma + s\frac{\pi}{\sigma\sqrt{6}}(y-f),\ s\in\{\pm1\}.$$

4.9 Laplace Likelihood

Laplace's Approximation

The following derivatives are needed:

$$\begin{aligned}
\ln p(y|f) &= -\ln(2b) - \frac{|f-y|}{b} \\
\frac{\partial \ln p}{\partial f} &= \frac{\operatorname{sign}(f-y)}{b} \\
\frac{\partial^2 \ln p}{(\partial f)^2} &= \frac{\partial^3 \ln p}{(\partial f)^3} = \frac{\partial^3 \ln p}{(\partial \ln \sigma_n)(\partial f)^2} = 0 \\
\frac{\partial \ln p}{\partial \ln \sigma_n} &= \frac{|f-y|}{b} - 1
\end{aligned}$$

Expectation Propagation

Expectation propagation requires integration against a Gaussian measure for moment matching. We need to evaluate $\ln Z = \ln \int \mathcal{L}(y|f,\sigma_n^2)\mathcal{N}(f|\mu,\sigma^2)df$ as well as the derivatives $\frac{\partial \ln Z}{\partial \mu}$ and $\frac{\partial^2 \ln Z}{\partial \mu^2}$ where $\mathcal{N}(f|\mu,\sigma^2) = \frac{1}{\sqrt{2\pi\sigma^2}} \exp\left(-\frac{(f-\mu)^2}{2\sigma^2}\right)$, $\mathcal{L}(y|f,\sigma_n^2) = \frac{1}{2b}\exp\left(-\frac{|y-f|}{b}\right)$, and $b = \frac{\sigma_n}{\sqrt{2}}$. As a first step, we reduce the number of parameters by means of the substitution $\tilde{f} = \frac{f-y}{\sigma_n}$ yielding

$$\begin{aligned}
Z &= \int \mathcal{L}(y|f,\sigma_n^2)\mathcal{N}(f|\mu,\sigma^2)df \\
&= \frac{1}{\sqrt{2\pi}\sigma}\frac{\sqrt{2}}{2\sigma_n}\int \exp\left(-\frac{(f-\mu)^2}{2\sigma^2}\right)\exp\left(-\sqrt{2}\frac{|f-y|}{\sigma_n}\right)df \\
&= \frac{\sqrt{2}}{2\sigma\sqrt{2\pi}}\int \exp\left(-\frac{(\sigma_n\tilde{f}+y-\mu)^2}{2\sigma^2}\right)\exp\left(-\sqrt{2}|\tilde{f}|\right)d\tilde{f} \\
&= \frac{\sigma_n}{\sigma\sigma_n\sqrt{2\pi}}\int \exp\left(-\frac{\sigma_n^2\left(\tilde{f}-\frac{\mu-y}{\sigma_n}\right)^2}{2\sigma^2}\right)\mathcal{L}(\tilde{f}|0,1)d\tilde{f} \\
&= \frac{1}{\sigma_n}\int \mathcal{L}(f|0,1)\mathcal{N}(f|\tilde{\mu},\tilde{\sigma}^2)df \\
\ln Z &= \ln\tilde{Z} - \ln\sigma_n = \ln\int \mathcal{L}(f|0,1)\mathcal{N}(f|\tilde{\mu},\tilde{\sigma}^2)df - \ln\sigma_n
\end{aligned}$$

with $\tilde{\mu} = \frac{\mu - y}{\sigma_n}$ and $\tilde{\sigma} = \frac{\sigma}{\sigma_n}$. Thus, we concentrate on the simpler quantity $\ln \tilde{Z}$.

$$
\begin{aligned}
\ln Z &= \ln \int \exp\left(-\frac{(f-\tilde{\mu})^2}{2\tilde{\sigma}^2} - \sqrt{2}|f|\right) df \overbrace{- \ln \tilde{\sigma}\sqrt{2\pi} - \ln \sqrt{2}\sigma_n}^{C} \\
&= \ln \left[\int_{-\infty}^{0} \exp\left(-\frac{(f-\tilde{\mu})^2}{2\tilde{\sigma}^2} + \sqrt{2}f\right) df + \int_{0}^{\infty} \exp\left(-\frac{(f-\tilde{\mu})^2}{2\tilde{\sigma}^2} - \sqrt{2}f\right) df\right] + C \\
&= \ln \left[\int_{-\infty}^{0} \exp\left(-\frac{f^2 - 2(\overbrace{\tilde{\mu} + \tilde{\sigma}^2\sqrt{2}}^{m_-})f + \tilde{\mu}^2}{2\tilde{\sigma}^2}\right) df + \int_{0}^{\infty} \exp\left(-\frac{f^2 - 2(\overbrace{\tilde{\mu} - \tilde{\sigma}^2\sqrt{2}}^{m_+})f + \tilde{\mu}^2}{2\tilde{\sigma}^2}\right) df\right] + C \\
&= \ln \left[\exp\left(\frac{m_-^2}{2\tilde{\sigma}^2}\right) \int_{-\infty}^{0} \exp\left(-\frac{(f-m_-)^2}{2\tilde{\sigma}^2}\right) df + \exp\left(\frac{m_+^2}{2\tilde{\sigma}^2}\right) \int_{0}^{\infty} \exp\left(-\frac{(f-m_+)^2}{2\tilde{\sigma}^2}\right) df\right] - \frac{\tilde{\mu}^2}{2\tilde{\sigma}^2} + C \\
&= \ln \left[\exp\left(\frac{m_-^2}{2\tilde{\sigma}^2}\right) \int_{-\infty}^{0} \mathcal{N}(f|m_-, \tilde{\sigma}^2) df + \exp\left(\frac{m_+^2}{2\tilde{\sigma}^2}\right) \left(1 - \int_{-\infty}^{0} \mathcal{N}(f|m_+, \tilde{\sigma}^2) df\right)\right] - \frac{\tilde{\mu}^2}{2\tilde{\sigma}^2} - \ln \sqrt{2}\sigma_n \\
&= \ln \left[\exp\left(\frac{m_-^2}{2\tilde{\sigma}^2}\right) \Phi\left(\frac{m_-}{\tilde{\sigma}}\right) - \exp\left(\frac{m_+^2}{2\tilde{\sigma}^2}\right) \Phi\left(\frac{m_+}{\tilde{\sigma}}\right) + \exp\left(\frac{m_+^2}{2\tilde{\sigma}^2}\right)\right] - \frac{\tilde{\mu}^2}{2\tilde{\sigma}^2} - \ln \sqrt{2}\sigma_n
\end{aligned}
$$

Here, $\Phi(z) = \int_{-\infty}^{z} \mathcal{N}(f|0,1) df$ denotes the cumulative Gaussian distribution. Finally, we have

$$
\begin{aligned}
\ln Z &= \ln \left[\exp\left(-\sqrt{2}\tilde{\mu}\right) \Phi\left(\frac{m_-}{\tilde{\sigma}}\right) + \exp\left(\sqrt{2}\tilde{\mu}\right) \Phi\left(-\frac{m_+}{\tilde{\sigma}}\right)\right] + \tilde{\sigma}^2 - \ln \sqrt{2}\sigma_n \\
&= \ln \left[\exp\left(\underbrace{\ln \Phi(-z_+) + \sqrt{2}\tilde{\mu}}_{a_+}\right) + \exp\left(\underbrace{\ln \Phi(z_-) - \sqrt{2}\tilde{\mu}}_{a_-}\right)\right] + \tilde{\sigma}^2 - \ln \sqrt{2}\sigma_n \\
&= \ln(e^{a_+} + e^{a_-}) + \tilde{\sigma}^2 - \ln \sqrt{2}\sigma_n
\end{aligned}
$$

where $z_+ = \frac{\tilde{\mu}}{\tilde{\sigma}} + \tilde{\sigma}\sqrt{2} = \frac{\mu - y}{\sigma} + \frac{\sigma}{\sigma_n}\sqrt{2}$, $z_- = \frac{\tilde{\mu}}{\tilde{\sigma}} - \tilde{\sigma}\sqrt{2} = \frac{\mu - y}{\sigma} - \frac{\sigma}{\sigma_n}\sqrt{2}$ and $\tilde{\mu} = \frac{\mu - y}{\sigma_n}$, $\tilde{\sigma} = \frac{\sigma}{\sigma_n}$.

Now, using $\frac{d}{d\theta} \ln \Phi(z) = \frac{1}{\Phi(z)} \frac{d}{d\theta} \Phi(z) = \frac{\mathcal{N}(z)}{\Phi(z)} \frac{dz}{d\theta}$ we tackle first derivative

$$
\begin{aligned}
\frac{\partial \ln Z}{\partial \mu} &= \frac{e^{a_+} \frac{\partial a_+}{\partial \mu} + e^{a_-} \frac{\partial a_-}{\partial \mu}}{e^{a_+} + e^{a_-}} \\
\frac{\partial a_+}{\partial \mu} &= \frac{\partial}{\partial \mu} \ln \Phi(-z_+) + \frac{\sqrt{2}}{\sigma_n} \\
&= -\frac{\mathcal{N}(-z_+)}{\sigma \Phi(-z_+)} + \frac{\sqrt{2}}{\sigma_n} = -\frac{q_+}{\sigma} + \frac{\sqrt{2}}{\sigma_n} \\
\frac{\partial a_-}{\partial \mu} &= \frac{\partial}{\partial \mu} \ln \Phi(z_-) - \frac{\sqrt{2}}{\sigma_n} \\
&= \frac{\mathcal{N}(z_-)}{\sigma \Phi(z_-)} - \frac{\sqrt{2}}{\sigma_n} = \frac{q_-}{\sigma} - \frac{\sqrt{2}}{\sigma_n} \\
\frac{\partial a_\pm}{\partial \mu} &= \mp \frac{q_\pm}{\sigma} \pm \frac{\sqrt{2}}{\sigma_n}.
\end{aligned}
$$

as well as the second derivative

$$
\begin{aligned}
\frac{\partial^2 \ln Z}{\partial \mu^2} &= \frac{\frac{\partial}{\partial \mu}\left(e^{a_+}\frac{\partial a_+}{\partial \mu}\right) + \frac{\partial}{\partial \mu}\left(e^{a_-}\frac{\partial a_-}{\partial \mu}\right)}{e^{a_+} + e^{a_-}} - \left(\frac{\partial \ln Z}{\partial \mu}\right)^2 \\
\frac{\partial}{\partial \mu}\left(e^{a_\pm}\frac{\partial a_\pm}{\partial \mu}\right) &= e^{a_\pm}\left[\left(\frac{\partial a_\pm}{\partial \mu}\right)^2 + \frac{\partial^2 a_\pm}{\partial \mu^2}\right] \\
\frac{\partial^2 a_+}{\partial \mu^2} &= -\frac{1}{\sigma}\frac{\frac{\partial}{\partial \mu}\mathcal{N}(-z_+)\Phi(-z_+) - \frac{\partial}{\partial \mu}\Phi(-z_+)\mathcal{N}(-z_+)}{\Phi^2(-z_+)} \\
&= -\frac{1}{\sigma}\frac{\mathcal{N}(-z_+)\Phi(-z_+)\frac{\partial -z_+^2/2}{\partial \mu} - \mathcal{N}^2(-z_+)\frac{\partial -z_+}{\partial \mu}}{\Phi^2(-z_+)} \\
&= \frac{\mathcal{N}(-z_+)}{\sigma^2}\cdot\frac{\Phi(-z_+)z_+ - \mathcal{N}(-z_+)}{\Phi^2(-z_+)} = -\frac{q_+^2 - q_+ z_+}{\sigma^2} \\
\frac{\partial^2 a_-}{\partial \mu^2} &= \frac{1}{\sigma}\frac{\frac{\partial}{\partial \mu}\mathcal{N}(z_-)\Phi(z_-) - \frac{\partial}{\partial \mu}\Phi(z_-)\mathcal{N}(z_-)}{\Phi^2(z_-)} \\
&= \frac{1}{\sigma}\frac{\mathcal{N}(z_-)\Phi(z_-)\frac{\partial -z_-^2/2}{\partial \mu} - \mathcal{N}^2(z_-)\frac{\partial z_-}{\partial \mu}}{\Phi^2(z_-)} \\
&= \frac{\mathcal{N}(z_-)}{\sigma^2}\cdot\frac{-\Phi(z_-)z_- - \mathcal{N}(z_-)}{\Phi^2(z_-)} = -\frac{q_-^2 + q_- z_-}{\sigma^2} \\
\frac{\partial^2 a_\pm}{\partial \mu^2} &= -\frac{q_\pm^2 \mp q_\pm z_\pm}{\sigma^2}
\end{aligned}
$$

which can be simplified to

$$
\frac{\partial^2 \ln Z}{\partial \mu^2} = \frac{e^{a_+}b_+ + e^{a_-}b_-}{e^{a_+} + e^{a_-}} - \left(\frac{\partial \ln Z}{\partial \mu}\right)^2
$$

using

$$
\begin{aligned}
b_\pm = \left(\frac{\partial a_\pm}{\partial \mu}\right)^2 + \frac{\partial^2 a_\pm}{\partial \mu^2} &= \left(\mp\frac{q_\pm}{\sigma} \pm \frac{\sqrt{2}}{\sigma_n}\right)^2 - \frac{q_\pm^2 \mp q_\pm z_\pm}{\sigma^2} \\
&= \left(\frac{q_\pm}{\sigma} - \frac{\sqrt{2}}{\sigma_n}\right)^2 - \frac{q_\pm^2}{\sigma^2} \pm \frac{q_\pm z_\pm}{\sigma^2} \\
&= \frac{2}{\sigma_n^2} - \left(\frac{\sqrt{8}}{\sigma\sigma_n} \mp \frac{z_\pm}{\sigma^2}\right)q_\pm.
\end{aligned}
$$

We also need

$$
\frac{\partial \ln Z}{\partial \ln \sigma_n} = \frac{e^{a_+}\frac{\partial a_+}{\partial \ln \sigma_n} + e^{a_-}\frac{\partial a_-}{\partial \ln \sigma_n}}{e^{a_+} + e^{a_-}} - \frac{2\sigma^2}{\sigma_n^2} - 1.
$$

Variational Bayes

We need $h(\gamma)$ and its derivatives as well as $\beta(\gamma)$:

$$\begin{aligned}
h(\gamma) &= \frac{2}{\sigma_n^2}\gamma + \ln(2\sigma_n^2) + y^2\gamma^{-1} \\
h'(\gamma) &= \frac{2}{\sigma_n^2} - y^2\gamma^{-2} \\
h''(\gamma) &= 2y^2\gamma^{-3} \\
\beta(\gamma) &= y\gamma^{-1}
\end{aligned}$$

4.10 Student's t Likelihood

The likelihood has two hyperparameters (both represented in the log domain to ensure positivity): the degrees of freedom ν and the scale σ_n with mean y (for $\nu > 1$) and variance $\frac{\nu}{\nu-2}{\sigma_n}^2$ (for $\nu > 2$).

$$p(y|f) = Z \cdot \left(1 + \frac{(f-y)^2}{\nu\sigma_n^2}\right)^{-\frac{\nu+1}{2}}, \quad Z = \frac{\Gamma\left(\frac{\nu+1}{2}\right)}{\Gamma\left(\frac{\nu}{2}\right)\sqrt{\nu\pi\sigma_n^2}}$$

Laplace's Approximation

For the mode fitting procedure, we need derivatives up to third order; the hyperparameter derivatives at the mode require some mixed derivatives. All in all, using $r = y - f$, we have

$$\begin{aligned}
\ln p(y|f) &= \ln\Gamma\left(\frac{\nu+1}{2}\right) - \ln\Gamma\left(\frac{\nu}{2}\right) - \frac{1}{2}\ln\nu\pi\sigma_n^2 - \frac{\nu+1}{2}\ln\left(1 + \frac{r^2}{\nu\sigma_n^2}\right) \\
\frac{\partial \ln p}{\partial f} &= (\nu+1)\frac{r}{r^2+\nu\sigma_n^2} \\
\frac{\partial^2 \ln p}{(\partial f)^2} &= (\nu+1)\frac{r^2-\nu\sigma_n^2}{(r^2+\nu\sigma_n^2)^2} \\
\frac{\partial^3 \ln p}{(\partial f)^3} &= 2(\nu+1)\frac{r^3-3r\nu\sigma_n^2}{(r^2+\nu\sigma_n^2)^3} \\
\frac{\partial \ln p}{\partial \ln \nu} &= \frac{\partial Z}{\partial \ln\nu} - \frac{\nu}{2}\ln\left(1+\frac{r^2}{\nu\sigma_n^2}\right) + \frac{\nu+1}{2}\cdot\frac{r^2}{r^2+\nu\sigma_n^2} \\
\frac{\partial Z}{\partial \ln\nu} &= \frac{\nu}{2}\frac{d\ln\Gamma\left(\frac{\nu+1}{2}\right)}{d\ln\nu} - \frac{\nu}{2}\frac{d\ln\Gamma\left(\frac{\nu}{2}\right)}{d\ln\nu} - \frac{1}{2} \\
\frac{\partial^3 \ln p}{(\partial\ln\nu)(\partial f)^2} &= \nu\frac{r^2(r^2-3(\nu+1){\sigma_n}^2)+\nu\sigma_n^2}{(r^2+\nu\sigma_n^2)^3} \\
\frac{\partial \ln p}{\partial \ln \sigma_n} &= (\nu+1)\frac{r^2}{r^2+\nu\sigma_n^2} - 1 \\
\frac{\partial^3 \ln p}{(\partial\ln\sigma_n)(\partial f)^2} &= 2\nu\sigma_n^2(\nu+1)\frac{\nu\sigma_n^2-3r^2}{(r^2+\nu\sigma_n^2)^3}
\end{aligned}$$

4.11 Cumulative Logistic Likelihood

The likelihood has one hyperparameter (represented in the log domain), namely the standard deviation σ_n

$$p(y|f) = Z \cdot \cosh^{-2}(\tau(f-y)), \ \tau = \frac{\pi}{2\sigma_n\sqrt{3}}, \ Z = \frac{\pi}{4\sigma_n\sqrt{3}}$$

The following derivatives are needed where $\phi(x) \equiv \ln(\cosh(x))$

$$\begin{aligned}
\ln p(y|f) &= \ln(\pi) - \ln(4\sigma_n\sqrt{3}) - 2\phi\left(\tau(f-y)\right) \\
\frac{\partial \ln p}{\partial f} &= 2\tau\phi'\left(\tau(f-y)\right) \\
\frac{\partial^2 \ln p}{(\partial f)^2} &= -2\tau^2\phi''\left(\tau(f-y)\right) \\
\frac{\partial^3 \ln p}{(\partial f)^3} &= 2\tau^3\phi'''\left(\tau(f-y)\right) \\
\frac{\partial^3 \ln p}{(\partial \ln \sigma_n)(\partial f)^2} &= 2\tau^2\left(2\phi''\left(\tau(f-y)\right) + \tau(f-y)\phi'''\left(\tau(f-y)\right)\right) \\
\frac{\partial \ln p}{\partial \ln \sigma_n} &= 2\tau(f-y)\phi'\left(\tau(f-y)\right) - 1
\end{aligned}$$

4.12 GLM Likelihoods: Poisson, Negative Binomial, Weibull, Gamma, Exponential, Inverse Gaussian and Beta

Data y from a space other than $\mathbb{R}$ e.g. $\mathbb{N}$, $\mathbb{R}_+$ or $[0,1]$ can be modeled using generalised linear model likelihoods $p(y|f)$ where the expected value $\mathbb{E}[y] = \mu$ is related to the underlying Gaussian process f by means of an inverse link function $\mu = g(f)$. Typically, the likelihoods are from an exponential family, hence the variance $\mathbb{V}[y] = v(\mu)$, is a simple function of the mean μ as well as higher order moments such as skewness $\mathbb{S}[y] = s(\mu)$ and kurtosis $\mathbb{K}[y] = k(\mu)$.

Here, we directly specify the inverse link function $\mu = g(f)$ defining the mapping from the GP f to the mean intensity μ. For numerical reasons, we work with the log of the inverse link function $h(f) = \ln g(f)$ and use its derivatives h', h'' and h''' for subsequent computations. In the table below, we have summarised the GLM likelihood expressions, the moments, the range of their variables and the applicable inverse link functions.

Likelihood	$\rho =$	$v(\mu) =$	$s(\mu) =$	$k(\mu) =$	$p(y \mid f) =$	$y \in$	$\mu \in$	Inverse Links
Poisson	$\emptyset$	μ	$1/\sqrt{\mu}$	$1/\mu$	$\mu^y \exp(-\mu)/y!$	$\mathbb{N}$	$\mathbb{R}_+$	`exp`, `logistic*`
Neg. Binomial	$\{\ln r\}$	$\mu(\mu/r+1)$	$\sqrt{\frac{4(r+\mu)}{r\mu}} - \sqrt{\frac{r}{\mu(\mu+r)}}$	$\frac{6}{r} + \frac{r}{\mu(\mu+r)}$	$\binom{y+r-1}{y} r^r \mu^y/(r+\mu)^{r+y}$	$\mathbb{N}$	$\mathbb{R}_+$	`exp`, `logistic*`
Weibull	$\{\ln \kappa\}$	$\mu^2(\gamma_2/\gamma_1^2 - 1)$	$\frac{\gamma_3 - 3\gamma_1\gamma_2 + 2\gamma_1^3}{(\gamma_2-\gamma_1^2)^{3/2}}$	$\frac{\gamma_4 - 4\gamma_1\gamma_3 + 12\gamma_1^2\gamma_2 - 3\gamma_2^2 - 6\gamma_1^4}{(\gamma_2-\gamma_1^2)^2}$	$\kappa\gamma_1/\mu\,(y\gamma_1/\mu)^{\kappa-1}\exp\left(-(y\gamma_1/\mu)^\kappa\right)$	$\mathbb{R}_+\backslash\{0\}$	$\mathbb{R}_+\backslash\{0\}$	`exp`, `logistic*`
Gamma	$\{\ln \alpha\}$	μ^2/α	$2/\sqrt{\alpha}$	$6/\alpha$	$\frac{\alpha^\alpha y^{\alpha-1}}{\Gamma(\alpha)}\mu^{-\alpha}\exp\left(-\frac{y\alpha}{\mu}\right)$	$\mathbb{R}_+\backslash\{0\}$	$\mathbb{R}_+\backslash\{0\}$	`exp`, `logistic*`
Exponential	$\emptyset$	μ^2	2	6	$\mu^{-1}\exp\left(-\frac{y}{\mu}\right)$	$\mathbb{R}_+\backslash\{0\}$	$\mathbb{R}_+\backslash\{0\}$	`exp`, `logistic*`
Inv. Gauss	$\{\ln \lambda\}$	μ^3/λ	$3\sqrt{\mu/\lambda}$	$15\mu/\lambda$	$\sqrt{\frac{\lambda}{2\pi y^3}}\exp\left(-\frac{\lambda(y-\mu)^2}{2\mu^2 y}\right)$	$\mathbb{R}_+\backslash\{0\}$	$\mathbb{R}_+\backslash\{0\}$	`exp`, `logistic*`
Log Gauss	$\{\ln \sigma_n\}$	$(e^{\sigma_n^2}-1)\mu^2$	$(e^{\sigma_n^2}+2)\sqrt{e^{\sigma_n^2}-1}$	$e^{4\sigma_n^2} + 2e^{3\sigma_n^2} + 3e^{2\sigma_n^2} - 6$	$\frac{1}{y\sigma_n\sqrt{2\pi}}\exp\left(-\frac{(\log\mu - \sigma_n^2/2 - \log y)^2}{2\sigma_n^2}\right)$	$\mathbb{R}_+\backslash\{0\}$	$\mathbb{R}_+\backslash\{0\}$	`exp`, `logistic*`
Beta	$\{\ln \phi\}$	$\mu(1-\mu)/(1+\phi)$	$\frac{(2-4\mu)(1+\phi)}{\sqrt{v(\mu)}(2+\phi)}$	$6\frac{(\phi+1)^2 - v(\mu)(5\phi+6)}{v(\mu)(\phi+2)(\phi+3)}$	$\frac{\Gamma(\phi)}{\Gamma(\mu\phi)\Gamma((1-\mu)\phi)} y^{\mu\phi-1}(1-y)^{(1-\mu)\phi-1}$	$[0,1]$	$[0,1]$	`expexp`, `logit`

4.12.1 Inverse Link Functions

Possible inverse link functions and their properties ($\cup$ convex, $\cap$ concave, $\uparrow$ monotone) are summarised below:

`util/glm_invlink_*`	$g(f) = \mu =$	$g : \mathbb{R} \to$	g is	$h(f) = \ln\mu =$	h is
`exp`	e^f	$\mathbb{R}_+$	$\cup,\uparrow$	f	$\cup,\cap,\uparrow$
`logistic`	$\ell(f) = \ln(1+e^f)$	$\mathbb{R}_+$	$\cup,\uparrow$	$\ln(\ln(1+e^f))$	$\cap,\uparrow$
`logistic2`	$\ell\left(f + af\ell(f)\right)$	$\mathbb{R}_+$	$\uparrow$	$\ln(\ell\left(f + af\ell(f)\right))$	$\cap,\uparrow$
`expexp`	$\exp(-e^{-f})$	$[0,1]$	$\uparrow$	$-e^{-f}$	$\cup,\uparrow$
`logit`	$1/(1+e^{-f})$	$[0,1]$	$\uparrow$	$-\ln(1+e^{-f})$	$\cup,\uparrow$

Please see `doc/usageLik.m` for how to specify the pair likelihood function and link function in GPML.

Exponential inverse link: `exp`

For $g(f) = e^f$ things are simple since $h(f) = f$, $h'(f) = 1$ and $h''(f) = h'''(f) = 0$.

Logistic inverse link: `logistic`

For $g(f) = \ln(1 + e^f)$ the derivatives of $h(f)$ are given by

$$
\begin{aligned}
h(f) &= \ln(\ln(1 + e^f)) \\
h'(f) &= \frac{1}{\ln(1 + e^f)} s(-f),\ s(f) = \frac{1}{1 + e^f},\ s'(f) = \frac{-e^f}{(1 + e^f)^2} = -s(-f)s(f) \\
h''(f) &= \frac{1}{\ln(1 + e^f)} \frac{e^{-f}}{(1 + e^{-f})^2} - \frac{1}{\ln^2(1 + e^f)} \frac{e^f}{1 + e^f} \frac{1}{1 + e^{-f}} \\
&= h'(f) \left[s(f) - h'(f)\right] \\
h'''(f) &= h''(f) \left[s(f) - h'(f)\right] + h'(f) \left[\frac{-e^f}{(1 + e^f)^2} - h''(f)\right] \\
&= h''(f) \left[s(f) - 2h'(f)\right] - h'(f)s(f)s(-f).
\end{aligned}
$$

Note that $g(f) = e^{h(f)} = \ln(1 + e^f)$ is convex and $h(f) = \ln(\ln(1 + e^f))$ with

$$
h''(f) = \frac{1}{\ln(1 + e^f)} \left(1 - \frac{e^f}{\ln(1 + e^f)}\right) \frac{1}{1 + e^f} \frac{1}{1 + e^{-f}} \leqslant 0
$$

is concave since $e^f \geqslant \ln(1 + e^f)$ for all $f \in \mathbb{R}$.

Twice logistic inverse link: `logistic2`

Note that $h(f) = \ln(\ell(f + af\ell(f)))$ is – according to Seeger et al.[9] – concave.

Double negative exponential inverse link: `expexp`

For $g(f) = \exp(-e^{-f})$ the derivatives of $h(f)$ are given by

$$
\begin{aligned}
h(f) &= -e^{-f} \\
h'(f) &= -h(f) \\
h''(f) &= h(f) \\
h'''(f) &= -h(f)
\end{aligned}
$$

[9] Bayesian Intermittent Demand Forecasting, NIPS, 2016

Logit regression inverse link: `logit`

For $g(f) = 1/(1+e^{-f})$ the derivatives of $h(f)$ can be computed using the logistic inverse link function $h_\ell(f)$ since $h(f) = f - \exp(h_\ell(f))$

$$\begin{aligned} h(f) &= f - e^{h_\ell(f)} \\ h'(f) &= 1 - e^{h_\ell(f)} h'_\ell(f) \\ h''(f) &= -e^{h_\ell(f)}[h'_\ell(f)^2 + h''_\ell(f)] = e^{h_\ell(f)} s_\ell(-f) s_\ell^2(f) \\ h'''(f) &= -e^{h_\ell(f)}[h'_\ell(f)^3 + 3h''_\ell(f)h'_\ell(f) + h'''_\ell(f)] \end{aligned}$$

4.12.2 Poisson Likelihood

Count data $y \in \mathbb{N}^n$ can be modeled in the GP framework using the Poisson distribution $p(y) = \mu^y e^{-\mu}/y!$ with mean/variance $\mathbb{E}[y] = \mathbb{V}[y] = \mu$, skewness $\mathbb{S}[y] = 1/\sqrt{\mu}$ and kurtosis $\mathbb{K}[y] = 1/\mu$ leading to the likelihood

$$\begin{aligned} p(y|f) &= \mu^y \exp(-\mu)/y!, \ \mu = g(f) \\ \Leftrightarrow \ln p(y|f) &= y \cdot \ln g(f) - g(f) - \ln \Gamma(y+1). \end{aligned}$$

For Laplace's method to work, we need the first three derivatives of the log likelihood $\ln p(y|f)$, where $h(f) = \ln g(f)$

$$\begin{aligned} \ln p(y|f) &= y \cdot h(f) - \exp(h(f)) - \ln \Gamma(y+1) \\ \frac{\partial}{\partial f} \ln p(y|f) &= h'(f)\,[y - \exp(h(f))] \\ \frac{\partial^2}{\partial f^2} \ln p(y|f) &= h''(f)\,[y - \exp(h(f))] - [h'(f)]^2 \exp(h(f)) \\ \frac{\partial^3}{\partial f^3} \ln p(y|f) &= h'''(f)\,[y - \exp(h(f))] - 3h'(f) \cdot h''(f) \exp(h(f)) - [h'(f)]^3 \exp(h(f)) \\ & h'''(f)\,[y - \exp(h(f))] - h'(f)[h'(f)^2 + 3h''(f)] \exp(h(f)). \end{aligned}$$

Note that if $\ln \mu = h(f)$ is concave and $\mu = g(f)$ is convex then the Poisson likelihood $p(y|f)$ is log-concave in f which is the case for both `exp` and `logistic`.

4.12.3 Weibull Likelihood

Nonnegative data $y \in \mathbb{R}_+$ such as time-to-failure can be modeled in the GP framework using the Weibull distribution $p(y) = \kappa/\lambda (y/\lambda)^{\kappa-1} e^{-(y/\lambda)^\kappa}$ with shape parameter $\kappa > 0$, scale parameter $\lambda > 0$, mean $\mathbb{E}[y] = \lambda\gamma_1 = \mu$ where $\gamma_j = \Gamma(1 + j/\kappa)$, variance $\mathbb{V}[y] = \lambda^2\gamma_2 - \mu^2 = \mu^2(\gamma_2/\gamma_1^2 - 1)$, skewness $\mathbb{S}[y] = (\gamma_3 - 3\gamma_1\gamma_2 + 2\gamma_1^3)/(\gamma_2 - \gamma_1^2)^{3/2}$ and kurtosis $\mathbb{K}[y] = (\gamma_4 - 4\gamma_1\gamma_3 + 12\gamma_1^2\gamma_2 - 3\gamma_2^2 - 6\gamma_1^4)/(\gamma_2 - \gamma_1^2)^2$. Using the substitution $\mu = \lambda\gamma_1 \Leftrightarrow 1/\lambda = \gamma_1/\mu$, we obtain

$$\begin{aligned} p(y|f) &= \gamma_1 \frac{\kappa}{\mu} \left(\gamma_1 \frac{y}{\mu}\right)^{\kappa-1} \exp\left(-\left(\gamma_1 \frac{y}{\mu}\right)^\kappa\right), \ \mu = g(f) > 0 \\ \Leftrightarrow \ln p(y|f) &= \ln\left(\gamma_1 \frac{\kappa}{\mu}\right) + (\kappa - 1)\ln\left(\gamma_1 \frac{y}{\mu}\right) - \left(\gamma_1 \frac{y}{\mu}\right)^\kappa. \end{aligned}$$

Note that the Weibull likelihood $p(y|f)$ is log-concave in f neither for the `exp` nor for the `logistic` inverse link.

4.12.4 Gamma Likelihood

Nonnegative data $y \in \mathbb{R}_+$ can be modeled in the GP framework using the Gamma distribution $p(y) = \theta^{-\alpha}/\Gamma(\alpha)y^{\alpha-1}e^{-y/\theta}$ with shape parameter $\alpha > 0$, scale parameter $\theta > 0$, mean $\mathbb{E}[y] = \alpha\theta = \mu$, variance $\mathbb{V}[y] = \alpha\theta^2 = \mu^2/\alpha$, skewness $\mathbb{S}[y] = 2/\sqrt{\alpha}$ and kurtosis $\mathbb{K}[y] = 6/\alpha$. Using the substitution $\mu = \alpha\theta \Leftrightarrow \alpha/\mu = 1/\theta$, we obtain

$$\begin{aligned} p(y|f) &= \frac{\alpha^\alpha y^{\alpha-1}}{\Gamma(\alpha)}\mu^{-\alpha}\exp\left(-\frac{y\alpha}{\mu}\right),\ \mu = g(f) > 0 \\ \Leftrightarrow \ln p(y|f) &= -\alpha\left(\ln\mu + \frac{y}{\mu}\right) - \ln Z_\alpha(y),\ \ln Z_\alpha(y) = \ln\Gamma(\alpha) - \alpha\ln\alpha + (1-\alpha)\ln y. \end{aligned}$$

Note that if $\ln\mu = h(f)$ was convex and $\mu = g(f)$ was concave then the Gamma likelihood $p(y|f)$ would be log-concave in f which is not the case for both `exp` and `logistic`.

4.12.5 Exponential Likelihood

Nonnegative data $y \in \mathbb{R}_+$ can be modeled in the GP framework using the Exponential distribution $p(y) = \theta^{-1}e^{-y/\theta}$ with scale parameter $\theta > 0$, mean $\mathbb{E}[y] = \theta = \mu$, variance $\mathbb{V}[y] = \mu^2$, skewness $\mathbb{S}[y] = 2$ and kurtosis $\mathbb{K}[y] = 6$. We obtain

$$\begin{aligned} p(y|f) &= \mu^{-1}\exp\left(-\frac{y}{\mu}\right),\ \mu = g(f) > 0 \\ \Leftrightarrow \ln p(y|f) &= -\ln\mu - \frac{y}{\mu}. \end{aligned}$$

Note that for `exp` (but not for `logistic`) the likelihood is log-concave. The exponential distribution corresponds to the Gamma distribution with $\alpha = 1$ and the Weibull distribution with $\kappa = 1$.

4.12.6 Inverse Gaussian Likelihood

Nonnegative data $y \in \mathbb{R}_+^n$ can be modeled in the GP framework using the Inverse Gaussian distribution $p(y) = \sqrt{\lambda/(2\pi y^3)}\exp(-\lambda(y-\mu)^2/(2\mu^2 y))$ with shape parameter $\lambda > 0$, mean parameter $\mu > 0$, mean $\mathbb{E}[y] = \mu$, variance $\mathbb{V}[y] = \mu^3/\lambda$, skewness $\mathbb{S}[y] = 3\sqrt{\mu/\lambda}$ and kurtosis $\mathbb{K}[y] = 15\mu/\lambda$. We obtain

$$\begin{aligned} p(y|f) &= \sqrt{\frac{\lambda}{2\pi y^3}}\exp\left(-\frac{\lambda(y-\mu)^2}{2\mu^2 y}\right),\ \mu = g(f) > 0 \\ \Leftrightarrow \ln p(y|f) &= -\frac{\lambda(y-\mu)^2}{2\mu^2 y} - \ln Z_\alpha(y),\ \ln Z_\alpha(y) = -\frac{1}{2}(\ln\lambda - \ln 2\pi y^3). \end{aligned}$$

The inverse Gaussian likelihood is in general not log-concace in f for both `exp` and `logistic`.

4.12.7 Log Gaussian Likelihood

Nonnegative data $y \in \mathbb{R}_+^n$ can be modeled in the GP framework using the Log-normal distribution $p(y) = \exp\left(-(\log\mu - \sigma_n^2/2 - \log y)^2/(2\sigma_n^2)\right)/(y\sigma_n\sqrt{2\pi})$ with scale parameter $\sigma_n > 0$, and mean parameter $\mu > 0$, mean $\mathbb{E}[y] = \mu$, variance $\mathbb{V}[y] = (e^{\sigma_n^2} - 1)\mu^2$, skewness $\mathbb{S}[y] = (e^{\sigma_n^2} + 2)\sqrt{e^{\sigma_n^2} - 1}$

and kurtosis $\mathbb{K}[y] = e^{4\sigma_n^2} + 2e^{3\sigma_n^2} + 3e^{2\sigma_n^2} - 6$. We obtain

$$\begin{aligned} p(y|f) &= \frac{1}{y\sigma_n\sqrt{2\pi}} \exp\left(-\frac{(\log\mu - \sigma_n^2/2 - \log y)^2}{2\sigma_n^2}\right), \ \mu = g(f) > 0 \\ \Leftrightarrow \ln p(y|f) &= -\frac{(\log\mu - \sigma_n^2/2 - \log y)^2}{2\sigma_n^2} - \ln Z(y),\ \ln Z(y) = y\sigma_n\sqrt{2\pi}. \end{aligned}$$

The log Gaussian likelihood is log-concace in f for the `exp` link function.

4.12.8 Beta Likelihood

Interval data $y \in [0,1]^n$ can be modeled in the GP framework using the Beta distribution $p(y) = y^{\alpha-1}(1-y)^{\beta-1}/B(\alpha,\beta)$ with shape parameters $\alpha,\beta > 0$, mean $\mathbb{E}[y] = \alpha/(\alpha+\beta)$ and variance $\mathbb{V}[y] = \alpha\beta/[(\alpha+\beta)^2(\alpha+\beta+1)]$ and $1/B(\alpha,\beta) = \Gamma(\alpha+\beta)/[\Gamma(\alpha)\Gamma(\beta)]$. Reparametrising using the mean parameter $\mu = \mathbb{E}[y] = \alpha/(\alpha+\beta)$, the shape parameter $\phi = \alpha+\beta$, the variance $\mathbb{V}[y] = \mu(1-\mu)/(1+\phi)$ and hence

$$\begin{aligned} p(y|f) &= \frac{\Gamma(\phi)}{\Gamma(\mu\phi)\Gamma((1-\mu)\phi)} y^{\mu\phi-1}(1-y)^{(1-\mu)\phi-1},\ \mu = g(f) > 0 \\ \Leftrightarrow \ln p(y|f) &= \ln\Gamma(\phi) - \ln\Gamma(\mu\phi) - \ln\Gamma((1-\mu)\phi) + (\mu\phi-1)\ln y + ((1-\mu)\phi-1)\ln(1-y). \end{aligned}$$

The Beta likelihood is in general not log-concace in f for both `exp` and `logistic`.

5 Mean Functions

A mean function $m_\phi : \mathcal{X} \to \mathbb{R}$ (with hyperparameters ϕ) of a GP f is a scalar function defined over the whole domain $\mathcal{X}$ that computes the expected value $m(x) = \mathbb{E}[f(x)]$ of f for the input x.

5.1 Interface

In the GPML toolbox, a mean function $m : \mathcal{X} \to \mathbb{R}$ needs to implement evaluation $m = m_\phi(X)$ and first derivatives $m_i = \frac{\partial}{\partial \phi_i} m$ with respect to the components i of the parameter $\phi \in \Phi$ as detailed below.

38 ⟨meanFunctions.m 38⟩≡

```
% Mean functions to be use by Gaussian process functions. There are two
% different kinds of mean functions: simple and composite:
%
% Simple mean functions:
%
%   meanZero       - zero mean function
%   meanOne        - one mean function
%   meanConst      - constant mean function
%   meanLinear     - linear mean function
%   meanPoly       - polynomial mean function
%   meanDiscrete   - precomputed mean for discrete data
%   meanGP         - predictive mean of another GP
%   meanGPexact    - predictive mean of a regression GP
%   meanNN         - nearest neighbor mean function
%   meanWSPC       - weighted sum of projected cosines
%
% Composite mean functions (see explanation at the bottom):
%
%   meanScale      - scaled version of a mean function
%   meanSum        - sum of mean functions
%   meanProd       - product of mean functions
%   meanPow        - power of a mean function
%   meanMask       - mask some dimensions of the data
%   meanPref       - difference mean for preference learning
%   meanWarp       - warped mean function
%
% Naming convention: all mean functions are named "mean/mean*.m".
%
%
% 1) With no or only a single input argument:
%
%    s = meanNAME  or  s = meanNAME(hyp)
%
% The mean function returns a string s telling how many hyperparameters hyp it
% expects, using the convention that "D" is the dimension of the input space.
% For example, calling "meanLinear" returns the string 'D'.
%
% 2) With two input arguments and one output argument:
%
%    m = meanNAME(hyp, x)
%
% The function computes and returns the mean vector m with components
% m(i) = m(x(i,:)) where hyp are the hyperparameters and x is an n by D matrix
% of data, where D is the dimension of the input space. The returned mean
```

```
% vector m is of size n by 1.
%
% 3) With two input arguments and two output arguments:
%
%    [m,dm] = meanNAME(hyp, x)
%
% The function computes and returns the mean vector m as in 2) above.
% In addition to that, the (linear) directional derivative function dm is
% returned. The call dhyp = dm(q) for a direction vector q of size n by 1
% returns a vector of directional derivatives dhyp = d (q'*m(x)) / d hyp of
% the same size as the hyperparameter vector hyp. The components of dhyp are
% defined as follows: dhyp(i) = q'*( d m(x) / d hyp(i) ).
%
% See also doc/usageMean.m.
%
⟨gpml copyright 6a⟩
```

5.2 Implemented Mean Functions

We offer simple and composite mean functions producing new mean functions $m(x)$ from existing mean functions $\mu_j(x)$. All code files are named according to the pattern `mean/mean<NAME>.m` for simple identification. This modular specification allows to define affine mean functions $m(x) = c + a^\top x$ or polynomial mean functions $m(x) = (c + a^\top x)^2$. All currently available mean functions are summarised in the following table.

Simple mean functions $m(x)$			
`<NAME>`	Mmeaning	$m(x) =$	ϕ
`Zero`	mean vanishes always	0	$\emptyset$
`One`	mean equals 1	1	$\emptyset$
`Const`	mean equals a constant	c	$c \in \mathbb{R}$
`Linear`	mean linearly depends on $x \in \mathcal{X} \subseteq \mathbb{R}^D$	$a^\top x$	$a \in \mathbb{R}^D$
`Poly`	mean polynomially depends on $x \in \mathcal{X} \subseteq \mathbb{R}^D$	$\sum_d a_d^\top x^d$	$a \in \mathbb{R}^{D\times d}$
`Discrete`	precomputed mean for discrete data $x \in \mathcal{X} \subseteq \mathbb{N}$	μ_x	$\mu \in \mathbb{R}^s$
`GP`	predictive mean of another GP	$\int y \cdot p(y\|\mathcal{D}, x)dy$	$\emptyset$
`GPexact`	predictive mean of a regression GP	$\int y \cdot p(y\|\mathcal{D}, x)dy$	ρ, ψ, σ_n
`NN`	nearest neighbor for a set $(z_j, m_j) \in \mathcal{X} \times \mathbb{R}$	$m_i,\ i = \arg\min_j d(x, z_j)$	$\emptyset$
`WSPC`	weighted sum of d projected cosines $x \in \mathcal{X} \subseteq \mathbb{R}^D$	$a^\top \cos(Wx + b)$	$W \in \mathbb{R}^{d\times D}$, $a, b \in \mathbb{R}^d$

Composite mean functions $[\mu_1(x), \mu_2(x), ..] \mapsto m(x)$			
`<NAME>`	meaning	$m(x) =$	ϕ
`Scale`	scale a mean	$\alpha\mu(x)$	$\alpha \in \mathbb{R}$
`Sum`	add up mean functions	$\sum_j \mu_j(x)$	$\emptyset$
`Prod`	multiply mean functions	$\prod_j \mu_j(x)$	$\emptyset$
`Pow`	raise a mean to a power	$\mu(x)^d$	$\emptyset$
`Mask`	act on components $I \subseteq [1, 2, .., D]$ of $x \in \mathcal{X} \subseteq \mathbb{R}^D$ only	$\mu(x_I)$	$\emptyset$
`Pref`	preference learning mean $x = [x_1; x_2]$, $x_i \subseteq \mathbb{R}^{D/2}$	$\mu(x_1) - \mu(x_2)$	$\emptyset$
`Warp`	warped mean	$g[\mu(x)]$	$\emptyset$

5.3 Usage of Implemented Mean Functions

Some code examples taken from `doc/usageMean.m` illustrate how to use simple and composite mean functions to specify a GP model.

Syntactically, a mean function `mf` is defined by

```
mn := 'func' | @func // simple
```

```
mf := {mn} | {mn, {param, mf}} | {mn, {mf, .., mf}} // composite
```

i.e., it is either a string containing the name of a mean function, a pointer to a mean function or one of the former in combination with a cell array of mean functions and an additional list of parameters.

40 ⟨doc/usageMean.m 40⟩≡

```
% demonstrate usage of mean functions
%
% See also meanFunctions.m.
%
⟨gpml copyright 6a⟩
clear all, close all
n = 5; D = 2; x = randn(n,D);            % create a random data set

% set up simple mean functions
m0 = {'meanZero'};  hyp0 = [];      % no hyperparameters are needed
m1 = {'meanOne'};   hyp1 = [];      % no hyperparameters are needed
mc = {@meanConst};  hypc = 2;  % also function handles are possible
ml = {@meanLinear}; hypl = [2;3];               % m(x) = 2*x1 + 3*x2
mp = {@meanPoly,2}; hypp = [1;1;2;3];  % m(x) = x1+x2+2*x1^2+3*x2^2
mn = {@meanNN,[1,0; 0,1],[0.9,0.5]}; hypn = [];  % nearest neighbor
s = 12; hypd = randn(s,1);           % discrete mean with 12 hypers
md = {'meanDiscrete',s};
hyp.cov = [0;0]; hypg = [];                     % GP predictive mean
xt = randn(2*n,D); yt = sign(xt(:,1)-xt(:,2));      % training data
mg = {@meanGP,hyp,@infEP,@meanZero,@covSEiso,@likErf,xt,yt};
hype = [0;0; log(0.1)];             % regression GP predictive mean
xt = randn(2*n,D); yt = xt(:,1).*xt(:,2);           % training data
me = {@meanGPexact,@meanZero,@covSEiso,xt,yt};

% set up composite mean functions
msc = {'meanScale',{m1}};      hypsc = [3; hyp1];      % scale by 3
msu = {'meanSum',{m0,mc,ml}};  hypsu = [hyp0; hypc; hypl];    % sum
mpr = {@meanProd,{mc,ml}};     hyppr = [hypc; hypl];      % product
mpo = {'meanPow',3,msu};       hyppo = hypsu;         % third power
mask = [false,true];     % mask excluding all but the 2nd component
mma = {'meanMask',mask,ml};    hypma = hypl(mask);
mpf = {@meanPref,ml};          hyppf = 2;  % linear pref with slope
mwp = {@meanWarp,ml,@sin,@cos};hypwp = 2;           % sin of linear

% 0) specify mean function
% mean = md; hyp = hypd; x = randi([1,s],n,1);
% mean = mn; hyp = hypn;
% mean = mg; hyp = hypg;
mean = me; hyp = hype;
% mean = m0;  hyp = hyp0;
% mean = msu; hyp = hypsu;
% mean = mpr; hyp = hyppr;
% mean = mpo; hyp = hyppo;
% mean = mpf; hyp = hyppf;

% 1) query the number of parameters
feval(mean{:})

% 2) evaluate the function on x
feval(mean{:},hyp,x)

% 3) compute the derivatives w.r.t. to hyperparameter i
i = 2; feval(mean{:},hyp,x,i)
```

6 Covariance Functions

A covariance function $k_\psi : \mathcal{X} \times \mathcal{X} \to \mathbb{R}$ (with hyperparameters ψ) of a GP f is a scalar function defined over the whole domain $\mathcal{X}^2$ that computes the covariance $k(x, z) = \mathbb{V}[f(x), f(z)] = \mathbb{E}[(f(x) - m(x))(f(z) - m(z))]$ of f between the inputs x and z.

6.1 Interface

Again, the interface is simple since only evaluation of the full covariance matrix $K = k_\psi(X)$ and its derivatives $K_i = \frac{\partial}{\partial \psi_i} K$ as well as cross terms $k_* = k_\psi(X, x_*)$ and $k_{**} = k_\psi(x_*, x_*)$ for prediction are required.

41 ⟨covFunctions.m 41⟩≡

```
% Covariance functions to be use by Gaussian process functions. There are two
% different kinds of covariance functions: simple and composite:
%
% 1) Elementary and standalone covariance functions:
%   covZero        - zero covariance function
%   covEye         - unit covariance function
%   covOne         - unit constant covariance function
%   covDiscrete    - precomputed covariance for discrete data
%
% 2) Composite covariance functions:
%   covScale       - scaled version of a covariance function
%   covSum         - sums of covariance functions
%   covProd        - products of covariance functions
%   covMask        - mask some dimensions of the data
%   covPref        - difference covariance for preference learning
%   covPER         - make stationary covariance periodic
%   covADD         - additive covariance function
%   covWarp        - warp input to a covariance function
%
% 3) Mahalanobis distance based covariances and their modes
%   covMaha        - generic "mother" covariance
%    * eye         - unit length scale
%    * iso         - isotropic length scale
%    * ard         - automatic relevance determination
%    * prot        - (low-rank) projection in input space
%    * fact        - factor analysis covariance
%    * vlen        - spatially varying length scale
%   covGE          - Gamma exponential covariance
%   covMatern      - Matern covariance function with nu=1/2, 3/2 or 5/2
%   covPP          - piecewise polynomial covariance function (compact support)
%   covRQ          - rational quadratic covariance function
%   covSE          - squared exponential covariance function
%
% 4) Dot product based covariances and their modes
%   covDot         - generic "mother" covariance
%    * eye         - unit length scale
%    * iso         - isotropic length scale
%    * ard         - automatic relevance determination
%    * pro         - (low-rank) projection in input space
%    * fac         - factor analysis covariance
%   covLIN         - linear covariance function
%   covPoly        - polynomial covariance function
%
```

```
% 5) Time series covariance functions on the positive real line
%   covFBM        - fractional Brownian motion covariance
%   covULL        - underdamped linear Langevin process covariance
%   covW          - i-times integrated Wiener process covariance
%   covOU         - i-times integrated Ornstein-Uhlenbeck process covariance
%
% 6) Standalone covariances
%   covNNone      - neural network covariance function
%   covLINone     - linear covariance function with bias
%   covPeriodic   - smooth periodic covariance function (1d)
%   covPeriodicNoDC - as above but with zero DC component and properly scaled
%   covCos        - sine periodic covariance function (1d) with unit period
%   covGabor      - Gabor covariance function
%
% 7) Shortcut covariances assembled from library
%   covConst      - covariance for constant functions
%   covNoise      - independent covariance function (i.e. white noise)
%   covPERiso     - make isotropic stationary covariance periodic
%   covPERard     - make ARD stationary covariance periodic
%   covMaterniso  - Matern covariance function with nu=1/2, 3/2 or 5/2
%   covMaternard  - Matern covariance function with nu=1/2, 3/2 or 5/2 with ARD
%   covPPiso      - piecewise polynomial covariance function (compact support)
%   covPPard      - piecewise polynomial covariance function (compact support)
%   covRQiso      - isotropic rational quadratic covariance function
%   covRQard      - rational quadratic covariance function with ARD
%   covSEiso      - isotropic squared exponential covariance function
%   covSEisoU     - same as above but without latent scale
%   covSEard      - squared exponential covariance function with ARD
%   covSEvlen     - spatially varying lengthscale squared exponential
%   covSEproj     - projection squared exponential covariance function
%   covLINiso     - linear covariance function
%   covLINard     - linear covariance function with ARD
%   covGaborard   - Gabor covariance function with ARD
%   covGaborsio   - isotropic Gabor covariance function
%   covSM         - spectral mixture covariance function
%
% 8) Special purpose (approximation) covariance functions
%   apxSparse     - sparse approximation: to be used for large scale inference
%                   problems with inducing points aka FITC
%   apxGrid       - grid interpolation:   to be used for large scale inference
%                   problems with Kronecker/Toeplitz/BTTB covariance matrix
%
% The covariance functions are written according to a special convention where
% the exact behaviour depends on the number of input and output arguments
% passed to the function. If you want to add new covariance functions, you
% should follow this convention if you want them to work with the function gp.
% There are four different ways of calling the covariance functions:
%
% 1) With no (or one) input argument(s):
%
%    s = cov
%
% The covariance function returns a string s telling how many hyperparameters it
% expects, using the convention that "D" is the dimension of the input space.
% For example, calling "covRQard" returns the string '(D+2)'.
%
% 2) With two input arguments and one output argument:
%
```

```
%    K = cov(hyp, x) equivalent to K = cov(hyp, x, [])
%
% The function computes and returns the covariance matrix where hyp are
% the hyperparameters and x is an n by D matrix of cases, where
% D is the dimension of the input space. The returned covariance matrix is of
% size n by n.
%
% 3) With three input arguments and one output argument:
%
%    Kz = cov(hyp, x, z)
%    kx = cov(hyp, x, 'diag')
%
% The function computes test set covariances; kx is a vector of self covariances
% for the test cases in x (of length n) and Kz is an (n by nz) matrix of cross
% covariances between training cases x and test cases z.
%
% 4) With two output arguments:
%
%     [K,dK] = cov(hyp, x) equivalent to [K,dK] = cov(hyp, x, [])
%     [K,dK] = cov(hyp, x, z)
%     [K,dK] = cov(hyp, x, 'diag')
%
% The function computes and returns the covariances K as in 3) above.
% In addition to that, the (linear) directional derivative function dK is
% returned. The two possible calls dhyp = dK(Q) and [dhyp,dx] = dK(Q) for a
% direction Q of the same size as K are possible. The return arguments dhyp
% and dx are the directional derivatives dhyp = d trace(Q'*K) / d hyp and
% dx = d trace(Q'*K) / d x are of the same size as the hyperparameter
% vector hyp and the input data x, respectively. The components of dhyp and
% dx are defined as follows: dhyp(i) = trace(Q'*( d K / d hyp(i) ))
% and dx(i,j) = trace(Q'*( d K / d x(i,j) )).
%
% Covariance functions can be specified in two ways: either as a string
% containing the name of the covariance function or using a cell array. For
% example:
%
%   cov = 'covRQard';
%   cov = {'covRQard'};
%   cov = {@covRQard};
%
% are supported. Only the second and third form using the cell array can be used
% for specifying composite covariance functions, made up of several
% contributions. For example:
%
%        cov = {'covScale', {'covRQiso'}};
%        cov = {'covSum', {'covRQiso','covSEard','covNoise'}};
%        cov = {'covProd',{'covRQiso','covSEard','covNoise'}};
%        cov = {'covMask',mask,'covSEiso'}
%   q=1; cov = {'covPPiso',q};
%   d=3; cov = {'covPoly',d};
%        cov = {'covADD',{[1,2],'covSEiso'}};
%        cov = {@apxSparse, {@covSEiso}, u};  where u are the inducing inputs
%
% specifies a covariance function which is the sum of three contributions. To
% find out how many hyperparameters this covariance function requires, we do:
%
%   feval(cov{:})
%
```

```
% which returns the string '3+(D+1)+1' (i.e. the 'covRQiso' contribution uses
% 3 parameters, the 'covSEard' uses D+1 and 'covNoise' a single parameter).
%
% See also doc/usageCov.m.
%
⟨gpml copyright 6a⟩
```

6.2 Implemented Covariance Functions

Similarly to the mean functions, we provide a whole algebra of covariance functions $k : \mathcal{X} \times \mathcal{X} \to \mathbb{R}$ with the same generic name pattern cov/cov<NAME>.m as before.

Besides a long list of simple covariance functions, we also offer a variety of composite covariance functions as shown in the following table.

1) Elementary and standalone covariance functions $k(x, x')$			
<NAME>	meaning	$k(x,z) =$	$\boldsymbol{\psi}$
Zero	covariance vanishes always	0	$\emptyset$
Eye	unit additive measurement noise	$\delta(x-z)$	$\emptyset$
One	unit constant	1	$\emptyset$
Discrete	precomputed covariance for discrete data $x \in \mathcal{X} \subseteq \mathbb{N}$	$k_{xx'}$ where $K = L^\top L$ is Cholesky decomposition, $L \in \mathbb{R}^{s \times s}$	L
2) Composite covariance functions $[\kappa_1(x,z), \kappa_2(x,z), ..] \mapsto k(x,z)$			
<NAME>	meaning	$k(x,z) =$	$\boldsymbol{\psi}$
Scale	modulate covariance function by a scalar	$\sigma_f^2 \kappa(x,z)$	$\ln \sigma_f$
Scale	modulate covariance function depending on input	$\sigma_f(x)\kappa(x,z)\sigma_f(z)$	$\boldsymbol{\phi}_\ell$
Sum	add up covariance functions	$\sum_j \kappa_j(x,z)$	$\emptyset$
Prod	multiply covariance functions	$\prod_j \kappa_j(x,z)$	$\emptyset$
Mask	act on components $I \subseteq [1,2,..,D]$ of $x \in \mathcal{X} \subseteq \mathbb{R}^D$ only	$\kappa(x_I, z_I)$	$\emptyset$
Pref	preference learning covariance $x = [x_1; x_2]$, $x_i \subseteq \mathbb{R}^{D/2}$	$\kappa(x_1,z_1) + \kappa(x_2,z_2) - \kappa(x_1,z_2) - \kappa(x_2,z_1)$	$\emptyset$
PER	turn covariance into a periodic, $\mathcal{X} \subseteq \mathbb{R}^D$	$\kappa(u(x), u(z))\ ,\ u(x) = \begin{bmatrix} \sin 2\pi x_p \\ \cos 2\pi x_p \end{bmatrix},\ x_p = \begin{cases} x & \text{eye} \\ x/p & \text{iso} \\ x/p & \text{ard} \end{cases}$	$\boldsymbol{\psi}_u$
ADD	additive, $\mathcal{X} \subseteq \mathbb{R}^D$, index degree set $\mathcal{D} = \{1,..,D\}$	$\sum_{d \in \mathcal{D}} \sigma_{f_d}^2 \sum_{\lvert I \rvert = d} \prod_{i \in I} \kappa(x_i, z_i; \psi_i)$	$\{\psi_1, .., \psi_D, \ln \sigma_{f_1}, .., \ln \sigma_{f_{\lvert \mathcal{D} \rvert}}\}$
Warp	warp inputs to a covariance function	$\kappa(p(x), p(z))$, where $p : \mathbb{R}^D \to \mathbb{R}^{D_p}$	$\emptyset$
3) Covariance functions based on Mahalanobis distances $k(x,z) = k(r)$, $r^2 = (x-z)^\top P^{-1}(x-z)$, where $x, z \in \mathcal{X} \subseteq \mathbb{R}^D$			
Maha	shared generic "mother" covariance function	$k(r) =$	$\emptyset$
GE	gamma exponential	$\exp\left(-\lvert r \rvert^\gamma\right),\ \gamma \in (0,2]$	$\ln(\gamma/(2-\gamma))$
Matern	Matérn, $f_1(t) = 1$, $f_3(t) = 1+t$, $f_5(t) = f_3(t) + \frac{t^2}{3}$	$f_d(\sqrt{d}r)\exp(-\sqrt{d}r)$	$\emptyset$
PP	compact support, piecewise polynomial $f_v(r)$	$\max(0, 1-r)^{j+v} \cdot f_v(r)$	$\emptyset$
RQ	rational quadratic	$\left(1 + \frac{1}{2}r^2/\alpha\right)^{-\alpha}$	$\ln \alpha$
SE	squared exponential	$\exp\left(-r^2/2\right)$	$\emptyset$
Allowable mode parameter values for Mahalanibis distance covariances			
'eye'	unit lengthscale	$P = I$	$\emptyset$
'iso'	isotropic lengthscale	$P = \ell^2 I,\ \ell \in \mathbb{R}_+$	$\ln \ell$
'ard'	automatic relevance determination	$P = \boldsymbol{\Lambda}^2,\ \boldsymbol{\Lambda} = \mathtt{diag}(\boldsymbol{\lambda}),\ \boldsymbol{\lambda} \in \mathbb{R}_+^D$	$\ln \boldsymbol{\Lambda}$
'proj'	(low-rank) projection in input space	$P^{-1} = L^\top L$, $L \in \mathbb{R}^{d \times D}$	L
'fact'	factor analysis	$P^{-1} = L^\top L + \mathtt{diag}(f)$, $f \in \mathbb{R}^D$, $L \in \mathbb{R}^{d \times D}$	$\{L, \ln f\}$
'vlen'	spatially varying lengthscale,	$k_\ell(x,z) = \left(\frac{\ell(x)\ell(z)}{l^2(x,z)}\right)^{D/2} k\left(\frac{r^2}{l^2(x,z)}\right),\ l^2(x,z) = \frac{\ell^2(x)+\ell^2(z)}{2}$	$\boldsymbol{\phi}_\ell$
4) Covariance functions based on Euclidean dot products $k(x,z) = k(s)$, $s = x^\top P^{-1} z$, where $x, z \in \mathcal{X} \subseteq \mathbb{R}^D$			
Dot	shared generic "mother" covariance function	$k(s) =$	$\emptyset$
LIN	linear covariance function	s	$\emptyset$
Poly	polynomial covariance	$\sigma_f^2(s+c)^d$	$\ln c$
Allowable mode parameter values for Euclidean dot product covariances			
'eye'	unit lengthscale	$P = I$	$\emptyset$
'iso'	isotropic lengthscale	$P = \ell^2 I,\ \ell \in \mathbb{R}_+$	$\ln \ell$
'ard'	automatic relevance determination	$P = \boldsymbol{\Lambda}^2,\ \boldsymbol{\Lambda} = \mathtt{diag}(\boldsymbol{\lambda}),\ \boldsymbol{\lambda} \in \mathbb{R}_+^D$	$\ln \boldsymbol{\Lambda}$
'proj'	(low-rank) projection in input space	$P^{-1} = L^\top L$, $L \in \mathbb{R}^{d \times D}$	L
'fact'	factor analysis	$P^{-1} = L^\top L + \mathtt{diag}(f)$, $f \in \mathbb{R}^D$, $L \in \mathbb{R}^{d \times D}$	$\{L, \ln f\}$
5) Time series covariance functions $k(x,z) = k(x,z)$, where $x, z \in \mathcal{X} = \mathbb{R}_+$			
<NAME>	meaning	$k(x,z) =$	$\boldsymbol{\psi}$
FBM	fractional Brownian motion covariance with Hurst index h	$\frac{1}{2}\sigma_f^2\left[\lvert x \rvert^{2h} - \lvert x-z \rvert^{2h} + \lvert z \rvert^{2h}\right]$	$\{\ln \sigma_f, -\ln(1/h - 1)\}$
ULL	underdamped linear Langevin process covariance	$k(t) = a^2 e^{-\mu t}\left(\frac{\sin(\omega t)}{\omega} + \frac{\cos(\omega t)}{\mu}\right),\ t = \lvert x-z \rvert$	$\{\ln \mu, \ln \omega, \ln a\}$
W	i-times integrated Wiener process covariance, $i \in \{-1,..,3\}$	$\kappa_0(x,z) = \sigma_f^2 \min(x,z),\ \kappa_i(x,z) = \int_0^x \int_0^z \kappa_i(\tilde{x}, \tilde{z}) d\tilde{x} d\tilde{z}$	$\ln \sigma_f$
OU	i-times integrated Ornstein-Uhlenbeck process covariance, $i \in \{0,1\}$	$\kappa_0(x,z) = \sigma_f^2 \exp(-\frac{\lvert x-z \rvert}{\ell}) + (\sigma_{f_0}^2 - \sigma_f^2)\exp(-\frac{\lvert x+z \rvert}{\ell})$	$\{\ln \ell, \ln \sigma_f, \ln \sigma_{f_0}\}$
6) Standalone covariance functions			
<NAME>	meaning	$k(x,z) =$	$\boldsymbol{\psi}$
NNone	neural net, $\mathcal{X} \subseteq \mathbb{R}^D$	$\sigma_f^2 \sin^{-1}\left(x^\top z / \sqrt{(\ell^2 + x^\top x)(\ell^2 + z^\top z)}\right)$	$\{\ln \ell, \ln \sigma_f\}$
LINone	linear with bias, $\mathcal{X} \subseteq \mathbb{R}^D$	$(x^\top z + 1)/t^2$	$\ln t$
Periodic	periodic, $\mathcal{X} \subseteq \mathbb{R}$	$\sigma_f^2 \exp\left(-\frac{2}{\ell^2}\sin^2[\pi(x-z)/p]\right)$	$\{\ln \ell, \ln p, \ln \sigma_f\}$
PeriodicNoDC	periodic, rescaled, DC component removed, $\mathcal{X} \subseteq \mathbb{R}$	$\sigma_f^2 \frac{\kappa(x-z) - \frac{1}{\pi}\int_0^\pi \kappa(t)dt}{\kappa(0) - \frac{1}{\pi}\int_0^\pi \kappa(t)dt},\ \kappa(x-z) = \exp\left(-\frac{2}{\ell^2}\sin^2[\pi(x-z)/p]\right)$	$\{\ln \ell, \ln p, \ln \sigma_f\}$
Cos	periodic cosine, $\mathcal{X} \subseteq \mathbb{R}$	$\sigma_f^2 \cos\left(\pi(x-z)/p\right)$	$\{\ln p, \ln \sigma_f\}$
Gabor	Gabor function, $\mathcal{X} \subseteq \mathbb{R}^D$, $\boldsymbol{\lambda}, p \in \mathbb{R}_+^D$	$\exp\left(-\frac{1}{2}t^\top \boldsymbol{\Lambda}^{-2} t\right)\cos\left(2\pi t_p^\top 1\right),\ t_p = \begin{cases} t & \text{eye} \\ t/p & \text{iso} \\ t/p & \text{ard} \end{cases},\ t = x - z$	$\{\ln \boldsymbol{\lambda}, \boldsymbol{\psi}_p\}$

7) Shortcut covariance functions assembled from library $k(x, z)$			
`<NAME>`	Meaning	$k(x, z) =$	ψ
`Const`	covariance equals a constant	σ_f^2	$\ln \sigma_f$
`Noise`	additive measurement noise	$\sigma_f^2 \delta(x - z)$	$\ln \sigma_f$
`PERiso`	turn covariance into a periodic, $\mathcal{X} \subseteq \mathbb{R}^D$	$\kappa(u(x), u(z))$, $u(x) = [\sin x_p, \cos x_p]$, $x_p = 2\pi x/p$	$\ln p$
`PERard`	turn covariance into a periodic, $\mathcal{X} \subseteq \mathbb{R}^D$	$\kappa(u(x), u(z))$, $u(x) = [\sin x_p, \cos x_p]$, $x_p = 2\pi x/p$	$\{\ln p_1, .., \ln p_D\}$
`Materniso`	Matérn, $\mathcal{X} \subseteq \mathbb{R}^D$, $f_1(t) = 1$, $f_3(t) = 1 + t$, $f_5(t) = f_3(t) + \frac{t^2}{3}$	$\sigma_f^2 f_d(r_d) \exp(-r_d)$, $r_d = \sqrt{\frac{d}{\ell^2}(x-z)^\top(x-z)}$	$\{\ln \ell, \ln \sigma_f\}$
`Maternard`	Matérn, $\mathcal{X} \subseteq \mathbb{R}^D$, $f_1(t) = 1$, $f_3(t) = 1 + t$, $f_5(t) = f_3(t) + \frac{t^2}{3}$	$\sigma_f^2 f_d(r_d) \exp(-r_d)$, $r_d = \sqrt{d(x-z)^\top \boldsymbol{\Lambda}^{-2}(x-z)}$	$\{\ln \lambda_1, .., \ln \lambda_D, \ln \sigma_f\}$
`PPiso`	compact support, piecewise polynomial $f_\nu(r)$	$\sigma_f^2 \max(0, 1-r)^{j+\nu} \cdot f_\nu(r)$, $r = \frac{\|x-z\|}{\ell}$, $j = \lfloor \frac{D}{2} \rfloor + \nu + 1$	$\{\ln \ell, \ln \sigma_f\}$
`PPard`	compact support, piecewise polynomial $f_\nu(r)$	$\sigma_f^2 \max(0, 1-r)^{j+\nu} \cdot f_\nu(r)$, $r^2 = (x-z)^\top \boldsymbol{\Lambda}^{-2}(x-z)$	$\{\ln \lambda_1, .., \ln \lambda_D, \ln \sigma_f\}$
`RQiso`	rational quadratic, $\mathcal{X} \subseteq \mathbb{R}^D$	$\sigma_f^2 \left(1 + \frac{1}{2\alpha\ell^2}(x-z)^\top(x-z)\right)^{-\alpha}$	$\{\ln \ell, \ln \sigma_f, \ln \alpha\}$
`RQard`	rational quadratic, $\mathcal{X} \subseteq \mathbb{R}^D$	$\sigma_f^2 \left(1 + \frac{1}{2\alpha}(x-z)^\top \boldsymbol{\Lambda}^{-2}(x-z)\right)^{-\alpha}$	$\{\ln \lambda_1, .., \ln \lambda_D, \ln \sigma_f, \ln \alpha\}$
`SEiso`	diagonal squared exponential, $\mathcal{X} \subseteq \mathbb{R}^D$	$\sigma_f^2 \exp\left(-\frac{1}{2\ell^2}(x-z)^\top(x-z)\right)$	$\{\ln \ell, \ln \sigma_f\}$
`SEisoU`	squared exponential, $\mathcal{X} \subseteq \mathbb{R}^D$	$\exp\left(-\frac{1}{2\ell^2}(x-z)^\top(x-z)\right)$	$\ln \ell$
`SEard`	automatic relevence determination squared exponential	$\sigma_f^2 \exp\left(-\frac{1}{2}(x-z)^\top \boldsymbol{\Lambda}^{-2}(x-z)\right)$	$\{\ln \lambda_1, .., \ln \lambda_D, \ln \sigma_f\}$
`SEvlen`	spatially varying lengthscale squared exponential	$\sigma_f^2 \left(\frac{a}{b}\right)^{\frac{D}{2}} \exp\left(-\frac{\|x-z\|^2}{b}\right)$, $a = 2\ell(x)\ell(z)$, $b = \ell^2(x) + \ell^2(z)$	$\{\boldsymbol{\phi}_\ell, \ln \sigma_f\}$
`SEproj`	factor analysis squared exponential	$\sigma_f^2 \exp\left(-\frac{1}{2}(x-z)^\top L^\top L(x-z)\right)$, $L \in \mathbb{R}^{d \times D}$	$\{L, \ln \sigma_f\}$
`LINard`	linear with diagonal weighting	$x^\top \boldsymbol{\Lambda}^{-2} z$	$\{\ln \lambda_1, .., \ln \lambda_D\}$
`LINiso`	linear with isotropic weighting	$x^\top z/\ell^2$	$\ln \ell$
`Gaborard`	anisotropic Gabor function, $\mathcal{X} \subseteq \mathbb{R}^D$, $\boldsymbol{\lambda}, p \in \mathbb{R}_+^D$	$\exp\left(-\sum_{d=1}^D \frac{t_d^2}{2\lambda_d^2}\right) \cos\left(2\pi \sum_{d=1}^D t_d/p_d\right)$, $t_d = x_d - z_d$	$\{\ln \boldsymbol{\lambda}, \ln p\}$
`Gaboriso`	isotropic Gabor function, $\mathcal{X} \subseteq \mathbb{R}^D$, $\ell, p \in \mathbb{R}_+$	$\exp(-\frac{t^\top t}{2\ell^2}) \cos(2\pi t^\top 1/p)$, $t = x - z$	$\{\ln \ell, \ln p\}$
`SM`	spectral mixture, $\mathcal{X} \subseteq \mathbb{R}^D$, $w \in \mathbb{R}_+^Q$, $M, V \in \mathbb{R}_+^{D \times Q}$	$w^\top \left(\exp(-\frac{1}{2}V^\top t^2) \odot \cos(M^\top t)\right)$, $t = 2\pi(x - z)$	$\{\ln w, \ln M, \ln V\}$
	spectral mixture product, $\mathcal{X} \subseteq \mathbb{R}^D$, $W \in \mathbb{R}_+^{D \times Q}$, $M, V \in \mathbb{R}_+^{D \times Q}$	$\prod_{d=1}^D w_d^\top \left(\exp(-\frac{1}{2}v_d t_d^2) \odot \cos(m_d t_d)\right)$, $t_d = 2\pi(x_d - z_d)$	$\{\ln W, \ln M, \ln V\}$

The spectral mixture covariance `covSM` was introduced by Wilson & Adams Gaussian Process Kernels for Pattern Discovery and Extrapolation, ICML, 2013.

The periodic covariance function `covPER` starts from a stationary covariance function that depends on the data only through a distance $r^2 = (x - x')^\top \boldsymbol{\Lambda}^{-2}(x - x')$ such as `covMatern`, `covPP`, `covRQ`, `covSE` and turns them into a periodic covariance function by embedding the data $x \in \mathbb{R}^D$ into a periodic high-dimensional space $x_p = u(x) \in \mathbb{R}^{2D}$ by a function $u(x) = 2\pi \text{diag}(p^{-1})x$.

The additive covariance function `covADD` starts from a one-dimensional covariance function $\kappa(x_i, x_i', \psi_i)$ acting on a single component $i \in [1, .., D]$ of x. From that, we define covariance functions $\kappa_I(x_I, x_I) = \prod_{i \in I} \kappa(x_i, x_i', \psi_i)$ acting on vector-valued inputs x_I. The sums of exponential size can efficiently be computed using the Newton-Girard formulae. Samples functions drawn from a GP with additive covariance are additive functions. The number of interacting variables $|I|$ is a measure of how complex the additive functions are.

6.3 Usage of Implemented Covariance Functions

Some code examples taken from `doc/usageCov.m` illustrate how to use simple and composite covariance functions to specify a GP model.

Syntactically, a covariance function `cf` is defined by

```
cv := 'func' | @func // simple

cf := {cv} | {cv, {param, cf}} | {cv, {cf, .., cf}} // composite
```

i.e., it is either a string containing the name of a covariance function, a pointer to a covariance function or one of the former in combination with a cell array of covariance functions and an additional list of parameters.

45 ⟨doc/usageCov.m 45⟩≡

```
% demonstrate usage of covariance functions
%
% See also covFunctions.m.
%
⟨gpml copyright 6a⟩
clear all, close all
n = 5; D = 3; x = randn(n,D); xs = randn(3,D);  % create a data set

```

```
% set up simple covariance functions
cn  = {'covNoise'}; sn = .1;  hypn = log(sn);  % one hyperparameter
cc  = {@covConst};   sf = 2;  hypc = log(sf); % function handles OK
ce  = {@covEye};              hype = [];                 % identity
cl  = {@covLIN};              hypl = []; % linear is parameter-free
cla = {'covLINard'}; L = rand(D,1); hypla = log(L);  % linear (ARD)
cli = {'covLINiso'}; l = rand(1);   hypli = log(l);    % linear iso
clo = {@covLINone}; ell = .9; hyplo = log(ell);  % linear with bias
cp  = {@covPoly,3}; c = 2; hypp = log([c;sf]);   % third order poly
cga = {@covSEard};   hypga = log([L;sf]);       % Gaussian with ARD
cgi = {'covSEiso'};  hypgi = log([ell;sf]);    % isotropic Gaussian
cgu = {'covSEisoU'}; hypgu = log(ell);   % isotropic Gauss no scale
cra = {'covRQard'}; al = 2; hypra = log([L;sf;al]); % ration. quad.
cri = {@covRQiso};          hypri = log([ell;sf;al]);   % isotropic
cma = {@covMaternard,5};  hypma = log([ell;sf]); % Matern class d=5
cmi = {'covMaterniso',3}; hypmi = log([ell;sf]); % Matern class d=3
cnn = {'covNNone'}; hypnn = log([L;sf]);           % neural network
cpe = {'covPeriodic'}; p = 2; hyppe = log([ell;p;sf]);   % periodic
cpn = {'covPeriodicNoDC'}; p = 2; hyppe = log([ell;p;sf]); % w/o DC
cpc = {'covCos'}; p = 2; hypcpc = log([p;sf]);         % cosine cov
cca = {'covPPard',3}; hypcc = hypgu;% compact support poly degree 3
cci = {'covPPiso',2}; hypcc = hypgi;% compact support poly degree 2
cgb = {@covGaboriso}; ell = 1; p = 1.2; hypgb=log([ell;p]); % Gabor
Q = 2; w = ones(Q,1)/Q; m = rand(D,Q); v = rand(D,Q);
csm = {@covSM,Q}; hypsm = log([w;m(:);v(:)]);    % Spectral Mixture
cvl = {@covSEvlen,{@meanLinear}}; hypvl = [1;2;1; 0]; % var lenscal
s = 12; cds = {@covDiscrete,s};      % discrete covariance function
L = randn(s); L = chol(L'*L); L(1:(s+1):end) = log(diag(L));
hypds = L(triu(true(s))); xd = randi([1,s],[n,1]); xsd = [1;3;6];
cfa = {@covSEfact,2}; hypfa = randn(D*2,1);       % factor analysis

% set up composite i.e. meta covariance functions
csc = {'covScale',cgu};      hypsc = [log(3); hypgu];  % scale by 9
csu = {'covSum',{cn,cc,cl}}; hypsu = [hypn; hypc; hypl];      % sum
cpr = {@covProd,{cc,cci}};   hyppr = [hypc; hypcc];       % product
mask = [0,1,0]; %   binary mask excluding all but the 2nd component
cma = {'covMask',mask,cgi{:}}; hypma = hypgi;
% isotropic periodic rational quadratic
cpi = {'covPERiso',{@covRQiso}};
% periodic Matern with ARD
cpa = {'covPERard',{@covMaternard,3}};
% additive based on SEiso using unary and pairwise interactions
cad = {'covADD',{[1,2],'covSEiso'}};
% preference covariance with squared exponential base covariance
cpr = {'covPref',{'covSEiso'}}; hyppr = [0;0];
xp = randn(n,2*D); xsp = randn(3,2*D);

% 0) specify a covariance function
% cov = cma; hyp = hypma;
% cov = cci; hyp = hypcc;
% cov = csm; hyp = hypsm;
cov = cds; hyp = hypds; x = xd; xs = xsd;
% cov = cfa; hyp = hypfa;
% cov = cvl; hyp = hypvl;
% cov = cpr; hyp = hyppr; x = xp; xs = xsp;

% 1) query the number of parameters
feval(cov{:})
```

```

% 2) evaluate the function on x
[K,dK] = feval(cov{:},hyp,x)

% 3) evaluate the function on x and xs to get cross-terms
[kss,dkss] = feval(cov{:},hyp,xs,'diag')
[Ks, dKs ]  = feval(cov{:},hyp,x,xs)
```

7 Hyperpriors

A hyperprior $p(\theta)$ with $\theta = [\rho, \phi, \psi]$ is a joint probability distribution over the likelihood hyperparameters ρ, the mean hyperparameters ϕ and the covariance hyperparameters ψ. We concentrate on factorial priors $p(\theta) = \prod_j p_j(\theta_j)$. Hyperpriors can be used to regularise the optimisation of the hyperparameters via the marginal likelihood $Z(\theta)$ so that $p(\theta)Z(\theta)$ is maximised instead. As we wish to perform unconstrained optimisation, we require (mainly) smooth hyperpriors with infinite support.

7.1 Interface

In the GPML toolbox, a prior distribution $p(\theta)$ needs to implement the evaluation of the log density $\ln p(\theta)$ and its first derivative $\frac{\partial}{\partial\theta} \ln p(\theta)$. In addition, we require sampling capabilities i.e. the generation of $\theta \sim p(\theta)$.

47 ⟨priorDistributions.m 47⟩≡

```
% prior distributions to be used for hyperparameters of Gaussian processes
% using infPrior.
% There are two different kinds of prior distributions: simple and composite:
%
% simple prior distributions:
%
%   priorGauss          - univariate Gaussian
%   priorLaplace        - univariate Laplace
%   priorT              - univariate Student's t
%
%   priorSmoothBox1     - univariate interval (linear decay in log domain)
%   priorSmoothBox2     - univariate interval (quadr. decay in log domain)
%
%   priorGamma          - univariate Gamma, IR+
%   priorWeibull        - univariate Weibull, IR+
%   priorInvGauss       - univariate Inverse Gaussian, IR+
%   priorLogNormal      - univariate Log-normal, IR+
%
%   priorClamped or     - fix hyperparameter to its current value by setting
%   priorDelta            derivatives to zero, no effect on marginal likelihood
%
%   priorGaussMulti     - multivariate Gauss
%   priorLaplaceMulti   - multivariate Laplace
%   priorTMulti         - multivariate Student's t
%
%   priorClampedMulti or - fix hyperparameter to its current value by setting
%   priorDeltaMulti        derivatives to zero, no effect on marginal likelihood
%
%   priorEqualMulti or   - make several hyperparameters have the same value by
%   priorSameMulti         same derivative, no effect on marginal likelihood
%
```

```
% composite prior distributions (see explanation at the bottom):
%
%   priorMix              - nonnegative mixture of priors
%   priorTransform        - prior on g(t) rather than t
%
% Naming convention: all prior distributions are named "prior/prior*.m".
%
%
% 1) With only a fixed input arguments:
%
%    r = priorNAME(par1,par2,parN)
%
% The function returns a random sample from the distribution for e.g.
% random restarts, simulations or optimisation initialisation.
%
% 2) With one additional input arguments:
%
%    [lp,dlp] = priorNAME(par1,par2,parN, t)
%
% The function returns the log density at location t along with its first
% derivative.
%
% See also doc/usagePrior.m, inf/infPrior.m.
%
⟨gpml copyright 6a⟩
```

7.2 Implemented Hyperpriors

All code files are named according to the pattern `prior/prior<NAME>.m` for simple identification. All currently available hyperpriors are summarised in the following table.

Simple hyperpriors $p(\theta)$			
Univariate hyperpriors defined over the whole reals with mean μ and variace σ^2			
`<NAME>`	Meaning	$p(\theta) =$	$\boldsymbol{\tau}$
`Gauss`	normally distributed hyperparameter $\theta \in \mathbb{R}$	$\frac{1}{\sigma\sqrt{2\pi}} \exp\left(-\frac{(\theta-\mu)^2}{2\sigma^2}\right)$	$\mu \in \mathbb{R},\ \sigma^2 \in \mathbb{R}_+$
`Laplace`	double exponentially hyperparameter $\theta \in \mathbb{R}$	$\frac{1}{2b} \exp\left(-\frac{(\theta-\mu)}{b}\right),\ b = \sigma/\sqrt{2}$	$\mu \in \mathbb{R},\ \sigma^2 \in \mathbb{R}_+$
`T`	Student's t distributed hyperparameter $\theta \in \mathbb{R}$	$\frac{\Gamma(\frac{\nu+1}{2})}{\Gamma(\frac{\nu}{2})} \frac{1}{\sqrt{(\nu-2)\pi\sigma}} \left(1 + \frac{(\theta-\mu)^2}{(\nu-2)\sigma^2}\right)^{-\frac{\nu+1}{2}}$	$\mu \in \mathbb{R},\ \sigma^2, \nu \in \mathbb{R}_+$
Univariate hyperpriors with effective bounded support but defined over the whole real line			
`SmoothBox1`	interval hyperparameter $\theta \in \mathbb{R}$ i.e. $\theta \stackrel{\approx}{\in} [a,b]$	$\frac{1-\exp(\eta(a-b))}{b-a} \cdot \frac{1}{1+\exp(-\eta(\theta-a))} \cdot \frac{1}{1+\exp(\eta(\theta-b))}$<br>$\mu = \frac{a+b}{2},\ \sigma^2 = \frac{w^2}{1-\exp(-2g)} \frac{1+\pi^2/g^2}{12},\ g = \frac{w\eta}{2},\ w = \lvert a-b \rvert$	$a \leqslant b \in \mathbb{R},\ \eta \in \mathbb{R}_+$
`SmoothBox2`	localised hyperparameter $\theta \in \mathbb{R}$ i.e. $\theta \stackrel{\approx}{\in} [a,b]$	$\frac{1}{(1/\eta+1)(b-a)} \begin{cases} \mathcal{N}(\theta \lvert a, \sigma_{ab}^2) & t < a \\ 1 & t \in [a,b],\ \sigma_{ab} = \frac{b-a}{\eta\sqrt{2\pi}} \\ \mathcal{N}(\theta \lvert a, \sigma_{ab}^2) & b < t \end{cases}$<br>$\mu = \frac{a+b}{2},\ \sigma^2 = \frac{w^2}{4} \frac{\eta^3/3+\eta^2+4\eta/\pi+2/\pi}{\eta^3+\eta^2},\ w = \lvert a-b \rvert$	$a \leqslant b \in \mathbb{R},\ \eta \in \mathbb{R}_+$
Univariate hyperpriors supported only over the positive reals			
`Gamma`	Gamma hyperparameter $\theta \in \mathbb{R}_+$	$\frac{1}{\Gamma(k)t^k} \exp(\frac{\theta}{t})\theta^{k-1}$	$k \in \mathbb{R}_+,\ t \in \mathbb{R}_+$
`Weibull`	Weibull hyperparameter $\theta \in \mathbb{R}_+$	$\frac{k}{\lambda} \left(\frac{\theta}{\lambda}\right)^{k-1} \exp\left(-(\frac{\theta}{\lambda})^k\right)$	$k \in \mathbb{R}_+,\ \lambda \in \mathbb{R}_+$
`InverseGauss`	inverse Gaussian hyperparameter $\theta \in \mathbb{R}_+$	$\frac{1}{\sqrt{2\pi\theta^3/\lambda}} \exp\left(-\frac{\lambda(\theta-\mu)^2}{2\mu^2\theta}\right)$	$k \in \mathbb{R}_+,\ \lambda \in \mathbb{R}_+$
`LogNormal`	log-normal hyperparameter $\theta \in \mathbb{R}_+$	$\mathcal{N}(\theta \lvert \mu, \sigma^2) = \frac{1}{\theta\sigma\sqrt{2\pi}} \exp\left(-\frac{(\ln\theta-\mu)^2}{2\sigma^2}\right)$	$\mu \in \mathbb{R},\ \sigma^2 \in \mathbb{R}_+$
Multivariate hyperpriors supported all over $\mathbb{R}^D$ with mean $\boldsymbol{\mu}$ and covariance $\boldsymbol{\Sigma}$			
`GaussMulti`	multivariate normal distribution $\boldsymbol{\theta} \in \mathbb{R}^D$	$\lvert 2\pi\boldsymbol{\Sigma} \rvert^{-\frac{1}{2}} \exp\left(-\frac{1}{2}(\boldsymbol{\theta}-\boldsymbol{\mu})^\top \boldsymbol{\Sigma}^{-1} (\boldsymbol{\theta}-\boldsymbol{\mu})\right)$	$\boldsymbol{\mu} \in \mathbb{R}^D,\ \boldsymbol{\Sigma} \in \mathbb{R}^{D \times D}$
`LaplaceMulti`	multivariate Laplace distribution $\boldsymbol{\theta} \in \mathbb{R}^D$	$\lvert \sqrt{2}\boldsymbol{\Sigma} \rvert^{-\frac{1}{2}} \exp\left(-\sqrt{2} \left\lVert L^{-1}(\boldsymbol{\theta}-\boldsymbol{\mu}) \right\rVert_1\right),\ L^\top L = \boldsymbol{\Sigma}$	$\boldsymbol{\mu} \in \mathbb{R}^D,\ \boldsymbol{\Sigma} \in \mathbb{R}^{D \times D}$
`TMulti`	multivariate Student's t distribution $\boldsymbol{\theta} \in \mathbb{R}^D$	$\lvert (\nu-2)\pi\boldsymbol{\Sigma} \rvert^{-\frac{1}{2}} \frac{\Gamma(\frac{\nu+D}{2})}{\Gamma(\frac{\nu}{2})} \left(1 + \frac{(\boldsymbol{\theta}-\boldsymbol{\mu})^\top \boldsymbol{\Sigma}^{-1} (\boldsymbol{\theta}-\boldsymbol{\mu})}{(\nu-2)}\right)^{-\frac{\nu+D}{2}}$	$\boldsymbol{\mu} \in \mathbb{R}^D,\ \boldsymbol{\Sigma} \in \mathbb{R}^{D \times D},\ \nu \in \mathbb{R}$
Improper hyperpriors used to fix the value of a particular hyperparameter			
`Delta`<br>`Clamped`	clamped hyperparameter $\theta = \theta_0 \in \mathbb{R}$	$\delta(\theta - \theta_0)$	$\emptyset$
`DeltaMulti`<br>`ClampedMulti`	clamped hyperparameter $\boldsymbol{\theta} = \boldsymbol{\theta}_0 \in \mathbb{R}^D$	$\delta(\boldsymbol{\theta} - \boldsymbol{\theta}_0)$	$\emptyset$
`SameMulti`<br>`EqualMulti`	same hyperparameter $\boldsymbol{\theta} = \theta_0 \mathbf{1} \in \mathbb{R}^D$	$\delta\left(\prod_{i=1}^D (\theta_0 - \theta_i)\right)$	$\emptyset$
Composite hyperpriors $[\pi_1(\theta), \pi_2(\theta), ..] \mapsto p(\theta)$			
`Transform`	prior distribution on $g(\theta)$ instead of θ	$\pi(g(\theta))$	$\{g\}$
`Mix`	mixture distribution	$\sum_i w_i \pi_i(\theta)$	$\{w\}$

The `priorSmoothBox2` is a Gauss-uniform sandwich obtained by complementing a uniform distribution on $[a, b]$ with two Gaussian halves at each side. The parameter η balances the probability mass between the constituents so that $\eta/(\eta + 1)$ is used for the box and $1/(\eta + 1)$ for the Gaussian sides. Its brother `priorSmoothBox1` is the product of two sigmoidal functions.

The `priorDelta` or equivalently `priorClamped` can be used to exclude some hyperparameters from the optimisation. Their values are clamped to θ_0 and the derivative vanishes. There are also multivariate counterparts `priorDeltaMulti` and `priorClampedMulti`.

7.3 Usage of Implemented Hyperpriors

Some code examples taken from `doc/usagePrior.m` illustrate how to use univariate, multivariate and composite priors on hyperparameters. Syntactically, a hyperprior `hp` is defined by

```
func := Dist                        // prior distributions in prior/
      | Clamped | Delta // predefined for fixing the hyperparameter

pr   := 'func'        | @func              // univariate hyperprior
      |  'funcMulti'  | @funcMulti       // multivariate hyperprior
```

```
hp   := {pr} | {pr, {param, hp}} | {pr, {hp, .., hp}} // composite
```

i.e., it is either a string containing the name of a hyperprior function, a pointer to a hyperprior function or one of the former in combination with a cell array of hyperprior functions and an additional list of parameters. Furthermore, we have multivariate hyperprior variants and 2 (equivalent) predefined hyperpriors allowing to exclude variables from optimisation.

50 ⟨doc/usagePrior.m 50⟩≡

```
% demonstrate usage of prior distributions
%
% See also priorDistributions.m.
%
⟨gpml copyright 6a⟩
clear all, close all

% 1) specify some priors
% a) univariate priors
mu = 1.0; s2 = 0.01^2; nu = 3;
pg = {@priorGauss,mu,s2};                                   % Gaussian prior
pl = {'priorLaplace',mu,s2};                                 % Laplace prior
pt = {@priorT,mu,s2,nu};                                 % Student's t prior
p1 = {@priorSmoothBox1,0,3,15};  % smooth box constraints lin decay
p2 = {@priorSmoothBox2,0,2,15};  % smooth box constraints qua decay
pd = {'priorDelta'}; % fix value of prior exclude from optimisation
pc = {@priorClamped};                                  % equivalent to above
lam = 1.05; k = 2.5;
pw = {@priorWeibull,lam,k};                                  % Weibull prior

% b) meta priors
pmx = {@priorMix,[0.5,0.5],{pg,pl}};        % mixture of two priors
g = @exp; dg = @exp; ig = @log;
ptr = {@priorTransform,g,dg,ig,pg};    % Gaussian in the exp domain

% c) multivariate priors
m = [1;2]; V = [2,1;1,2];
pG = {@priorGaussMulti,m,V};                             % 2d Gaussian prior
pD = {'priorDeltaMulti'};   % fix value of prior exclude from optim
pC = {@priorClampedMulti};                             % equivalent to above

% 2) evaluation
% pri = pt;   hp = randn(1,3);
% pri = pmx;  hp = randn(1,3);
% pri = ptr;  hp = randn(1,3);
pri = pG;   hp = randn(2,3);

% a) draw a sample from the prior
feval(pri{:})

% b) evaluate prior and derivative if requires
[lp,dlp] = feval(pri{:},hp)

% 3) comprehensive example
x = (0:0.1:10)'; y = 2*x+randn(size(x));   % generate training data
mean = {@meanSum,{@meanConst,@meanLinear}}; % specify mean function
cov = {@covSEiso}; lik = {@likGauss};  % specify covariance and lik
hyp.cov = [log(1);log(1.2)]; hyp.lik = log(0.9); hyp.mean = [2;3];
par = {mean,cov,lik,x,y}; mfun = @minimize; % input for GP function
```

```
% a) plain marginal likelihood optimisation (maximum likelihood)
im = @infExact;                                       % inference method
hyp_plain = feval(mfun, hyp, @gp, -10, im, par{:});      % optimise

% b) regularised optimisation (maximum a posteriori) with 1d priors
prior.mean = {pg;pc};  % Gaussian prior for first, clamp second par
prior.cov  = {p1;[]}; % box prior for first, nothing for second par
im = {@infPrior,@infExact,prior};                     % inference method
hyp_p1 = feval(mfun, hyp, @gp, -10, im, par{:});         % optimise

% c) regularised optimisation (maximum a posteriori) with Nd priors
prior = [];                                       % clear the structure
% multivariate Student's t prior on the first and second mean hyper
prior.multi{1} = {@priorTMulti,[mu;mu],diag([s2,s2]),nu,...
                  struct('mean',[1,2])};            % use hyper struct
% Equivalent shortcut (same mu and s2 for all dimensions)
prior.multi{1} = {@priorTMulti,mu,s2,nu,struct('mean',[1,2])};
% multivariate Gaussian prior jointly on 1st and 3rd hyper
prior.multi{2} = {@priorGaussMulti,[mu;mu],diag([s2,s2]),...
                  [1,3]};                % use unwrapped hyper vector
% Equivalent shortcut (same mu and s2 for all dimensions)
prior.multi{2} = {@priorGaussMulti,mu,s2,[1,3]};
im = {@infPrior,@infExact,prior};                     % inference method
hyp_pN = feval(mfun, hyp, @gp, -10, im, par{:});         % optimise

[any2vec(hyp), any2vec(hyp_plain), any2vec(hyp_p1), any2vec(hyp_pN)]
```
